# How partial phenotyping to reduce generation intervals can help to increase annual genetic gain in selected honeybee populations

**DOI:** 10.1038/s41437-025-00768-8

**Published:** 2025-05-22

**Authors:** Tristan Kistler, Evert W. Brascamp, Benjamin Basso, Florence Phocas, Piter Bijma

**Affiliations:** 1 Université Paris-Saclay, INRAE, AgroParisTech, GABI, 78350 Jouy-en-Josas, France; 2 UMT PrADE, 84914 Avignon, France; 3 Animal Breeding and Genomics, Wageningen University & Research, P.O. Box 338, 6700 AH Wageningen, The Netherlands; 4 INRAE, UR 406 Abeilles et Environnement, 84914 Avignon, France

**Keywords:** Agriculture, Animal breeding

## Abstract

Honeybee breeding is organized around annual cycles, following seasonal change. Generation intervals are thus commonly multiples of whole years. Most queens are generally raised during spring or early summer in temperate climates. A generation interval of 1 year limits phenotyping to early recordable traits, before the spring following queens’ births. Some traits, however, can only be recorded later, as is typically the case for total honey yield. Their recording on selection candidates thus increases the generation interval to at least 2 years, a common interval on the dam path. Using stochastic simulation, we investigated the impact of halving the dam generation interval and therefore recording only early traits on candidate dams. The generation interval on the sire path remained at 2 years with complete phenotyping. Breeding goals with varying weights on early and late traits were considered, as well as negative to positive genetic correlations between traits. The acceleration of the breeding scheme generally increased genetic gain for two-trait breeding goals, from 0% up to +47% after 20 years of selection. Although inbreeding rates per generation were slightly lower in the accelerated breeding scheme, inbreeding rates per year were significantly higher. This was due to the faster generation turnover (+33%) leading to 20–30% higher inbreeding coefficients (+0.04 to +0.07) after 20 years of selection. To avoid too high inbreeding, shortening the generation interval should be accompanied by strategies to limit inbreeding while still retaining most of the genetic gain, such as increasing the breeding nucleus size by relaxing selection intensity.

## Introduction

Beekeeping is facing major challenges, with global threats such as climate change, pesticides, and the spread of parasites and diseases, driving high colony losses (Steinhauer et al. 2018; Hristov et al. 2020; Insolia et al. 2022). As a result, colony replacement and queen rearing have become more intensive to replace lost or unproductive stock (Aureille 2014; Kouchner et al. 2019). Possibly, having to actively choose breeding queens instead of letting colonies spontaneously requeen has created awareness for selective breeding among beekeepers. Selection programs are emerging worldwide with a variety of breeding schemes. In these breeding schemes, bee breeders generally aim to improve a collection of traits simultaneously, focusing more or less on colony production, manageability, and resilience (Guichard et al. 2019; Maucourt et al. 2020; Hoppe et al. 2020; Kistler et al. 2024a). They generally aim to obtain enough genetic gains in both the short and long term while minimizing inbreeding rates to ensure the sustainability of their breeding programs.

Honeybee breeding is usually organized around annual cycles, following seasonal changes. As a result, generation intervals are typically constrained to multiples of 1 year, ranging from 1 to 4 years (Uzunov et al. 2022; Basso et al. 2024). Most queens are generally raised during spring or early summer in temperate climates. This is mainly because queens mate shortly after birth, and colonies most reliably produce abundant drones (males) in that period (Page 1981). They then store the semen in their spermatheca and will not mate anymore after the onset of egg-lay, a few days or weeks later. Mated queens can then be introduced in queen-less colonies, using various methods (Büchler et al. 2024). Performance testing can only start months later, once new queens have replaced all previous workers with their own descendance, which takes at least 42 days after the egg-lay onset of the new queen according to Büchler et al. (2024). A generation interval of 1 year thus limits phenotyping to early recordable traits, before the spring or early summer following queens’ birth. These early traits might include hygienic behavior (Büchler et al. 2020) linked to brood diseases; manageability traits (gentleness and calmness); first winter survival, winter brood interruption, winter feed consumption or early spring colony development; and possibly partial honey yield depending on floral resources. Complete honey yield, however, can generally only be obtained after the following mating period, when queens are over 1 year old. Other important traits, such as swarming tendency, sometimes show significant variability only in colonies with older queens (Uzunov et al. 2014). To allow for complete colony phenotyping of candidates, most breeding schemes thus use a 2-year generation interval in dams. However, some use partial phenotyping to reduce it to 1 year (Basso et al. 2024; Kistler et al. 2024a).

Due to the high mortality rates in honeybees, reduced generation intervals can increase selection intensity and genetic diversity by allowing selection of queens in a bigger candidate population before their second winter. In addition, shortening generation intervals is a well-known and powerful way to increase the expected annual genetic gain, which is inversely proportional to the generation interval when ignoring inbreeding and for a fixed selection accuracy (Dickerson and Hazel 1944). However, reducing the generation interval often decreases selection accuracy due to a shorter phenotyping period. Thus, annual genetic gain increases only if the benefit of shortening the generation interval outweighs the reduction in accuracy.

Predicting genetic gain and inbreeding is challenging using deterministic derivations, especially when considering the haplo-diploidy and polyandry in honeybees, as well as the complex colony phenotypes that result from two genetic effects expressed by two different generations (the queen and its worker group). Stochastic simulation can be a useful tool for comparing breeding strategies. In honeybees, it allows for the inclusion of honeybee specificities in addition to detailed representations of breeding populations, including limited population sizes, overlapping generations, and unbalanced sizes of sister groups due to random mortality. In addition, the effect of limited genetic connection across contemporary groups (apiaries and years) on genetic parameters’ and breeding values’ estimation can also be integrated (Clément et al. 2001). Stochastic simulations are therefore useful to assess both expectations and standard deviations of the genetic gain and inbreeding coefficients in bee breeding schemes.

Using stochastic simulation integrating bee specificities, Du et al. (2023) studied the shortening of the sire’s generation interval from 3 to 2 years. Assuming genetic parameters known, they showed that the reduction in the generation interval brought more genetic gain (about +12% after 20 years) but also a more than 2-fold increase in mean inbreeding coefficients and their rates per year. However, the reduction of the generation interval was studied with selection for a single trait that was recorded for all alternative schemes. Furthermore, their assumption of known genetic parameters is likely to have underestimated the impact of parameter estimation on the variability of results (Du et al. 2022a, b). Estimation of genetic parameters is particularly challenging in honeybees as queen and worker effects are statistically highly confounded and populations usually have breeding nuclei of only a few tens of breeding queens (Maucourt et al. 2020; Guichard et al. 2020; Basso et al. 2024; Kistler et al. 2024a).

In this study, we will assess by stochastic simulation the impact of reducing the dam’s generation interval from 2 to 1 year in a two-trait selection program involving an early and a late recorded trait. The reduction in the dam generation interval meant that only the early trait could be phenotyped for candidate dams, while for candidate sires, complete phenotyping (i.e., phenotyping of both early and late traits) was maintained with a 2-year generation interval. Results in terms of genetic gain and inbreeding will be compared with a breeding scheme maintaining complete phenotyping and a 2-year generation interval on both dam and sire paths. Various breeding goals and genetic correlations between the early and late traits, as well as between worker and queen effects, are covered.

## Methods

### Genetic model

Simulations implemented an infinitesimal genetic model with individual queens, drones, and worker groups, with two additive genetic effects affecting colony phenotypes ($y$): the queen genetic effect expressed by the colony’s queen (${a}_{{q}}^{{Q}}$) and the average worker genetic effect expressed by the colony’s worker group ($\bar{{a}_{{w}}^{{W}}}$) (Kistler et al. 2021):

$$y={a}_{{q}}^{{Q}}+\bar{{a}_{{w}}^{{W}}}+E+\varepsilon$$

where $E$ is an environmental apiary by year effect and ‘$\varepsilon$’ is a random residual term. Simulation of apiary effects is described further below.

Two traits were simulated: an early and a late-recordable trait. The main equations for the generation of base individuals’ breeding values (BVs) and of their inheritance are given in File S1.

The number of drones mating each queen (i.e., the polyandry level) was set to 8 as all values above 5 gave very similar results in previous simulations (Kistler et al. 2021; Du et al. 2024).

### Breeding goals

Breeding goals (*H*) were defined as weighted sums of true BVs for each trait, using 5 combinations of weights on both traits. The weight combinations varied from placing all the emphasis on the early trait to all the emphasis on the late trait in 25% increments. The resulting combinations were: (1:0), (0.75:0.25), (0.5:0.5), (0.25:0.75), and (0:1), where the first number is the weight put on the early trait and the second on the late trait. The weight applied to each trait was added as a subscript to H (e.g., *H*_0.25:0.75_). The BVs in *H* for worker and queen effects were summed together with the same weight. The selection index combined the estimated breeding values (EBVs) of each trait (or phenotypes in the Initialization phase, see further below) with the same weights as the corresponding breeding goal. EBVs for worker and queen effects on the same trait were summed before applying the weights in the selection index as in the breeding goals. Dams used to produce queens (from fertilized eggs) were selected based on their average offspring EBVs (i.e., the EBVs of their worker group), because the early mating of BQs is irreversible, so that offspring queens have the expected breeding value of their dam’s worker group (Brascamp and Bijma 2014; Brascamp et al. 2016). Sires used to produce drones (from unfertilized eggs) were selected based on their own EBVs (Brascamp and Bijma 2014; Brascamp et al. 2016).

### Simulation process

**Fig. 1:**
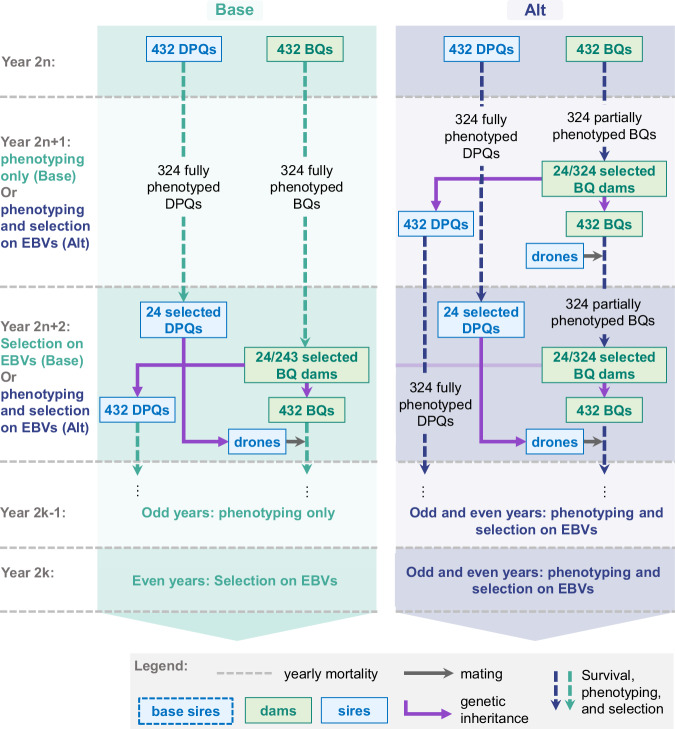
Simulation process of the Base breeding scheme, with a 2-year generation interval on dam and sire paths and complete phenotyping, and the Alternative breeding scheme, with a shortened dam generation interval of 1 year with partial phenotyping. BQ breeding queen, DPQ drone-producing queen. BQ and DPQ refers to queens that were candidates for selection, and “selected BQ” or “selected DPQs” to the selected dams and sires, respectively. Base: reference breeding scheme with complete phenotyping and a 2-years generation interval on both the dam and the sire path. Alt: accelerated breeding scheme in which only the early trait is phenotyped on potential dams, while potential sires are also phenotyped on the late trait. This partial phenotyping of the dams enables halving the dam generation interval to 1 year. Simulated mortality events randomly eliminate 25% of all selection candidates at the end of each year. See main text for a complete description of the simulation process. In year 2*n* + 2, in the Alt breeding scheme and because of overlapping generations, the new candidate DPQ offspring are not represented due to a lack of space, but are produced as in year 2*n* + 1.

Each simulation replicate was divided into three phases: an ‘Initialization’ phase, followed by a ‘Base breeding scheme’ (Base), and an ‘Alternative breeding scheme’ (Alt), both building upon the same Initialization outcomes (Fig. S1). The Initialization phase generated a closed population under selection and accumulated pedigrees and phenotypes for the subsequent phases. Base followed the same structure as Initialization with a generation interval of 2 years on both the dam and the sire path. In contrast, in Alt, the dam’s generation interval was reduced from 2 to 1 year, limiting phenotyping to the early trait only and resulting in an average generation interval of 1.5 years (25% less than in Base) (Fig. 1). The three phases will be detailed next.

#### Initialization phase

In the Initialization (Fig. S1), potential dams (BQs, breeding queens) and potential sires (DPQs, drone-producing queens) were phenotyped completely, i.e., tested on both the early and the late trait performance. Both dams and sires were selected and used for reproduction at 2 years old. In contrast to BQs, DPQs only genetically contribute drones to the next generations of breeding animals. Drones are born from unfertilized eggs, and thus, DPQs are often open-mated as this reduces costs (for example in Basso et al. 2024; Kistler et al. 2024a). Accordingly, in our simulations, DPQs were always open-mated to base drones. The Initialization phase lasted from year 1 to year 10 included. This duration ensured that at the moment of starting the following simulation phase: (1) the population was closed, (2) sufficient information was available for the first genetic parameter estimation that would follow, (3) it would not last too long so that inbreeding increases would be minimal. In year 1, 24 unrelated and non-inbred founder BQs were created by drawing their BVs directly from a multivariate normal distribution (see File S1 and section: “Genetic parameter sets and apiary effects”). These founder BQs were then mated to base drones, and produced worker groups to form colonies. In year 2, these colonies were phenotyped. Each BQ then produced 18 offspring BQs and 18 DPQs, which were open-mated to base drones and candidates for selection. Before colony phenotyping, 25% of all new BQs and 25% of all new DPQs were randomly eliminated to simulate mortality. In year 3 and each subsequent odd year, new BQ and DPQ colonies were phenotyped on the early and late trait, followed by a second random elimination of 25% of all colonies. The yearly mortality was simulated to reflect common high losses observed in reality (Potts et al. 2010; Seitz et al. 2022; Gray et al. 2023). These losses lead to imbalance in sister-group sizes compared to the planned design. Additionally, they reduce the number of selection candidates when they are selected in later years, impacting breeding results. As detailed previously, selection was for various alternative breeding goals where the weights for both traits differed. Among the 243 surviving colonies in each path, the best 24 BQ and the best 24 DPQ colonies were subjected to directional selection on a weighted sum of the phenotypes of both traits in standard deviation units. Each selected BQ produced 18 BQs and 18 DPQs in year 4 and each subsequent even year until year 10. Each of these offspring BQs, as all subsequent ones, were inseminated by drones produced by a single DPQ among the selected ones of that year, closing the breeding population. Each DPQ contributed to an equal number of matings. The choice of which DPQ to mate with each new BQ was random. In contrast, DPQs were always open-mated to base drones.

#### Base breeding scheme

Following this Initialization, in Base, BQs born in year 10 were selected in year 12 by directional selection based on the weighted sum of the estimated breeding values (EBVs) of both traits. Everything else remained the same as in the Initialization phase (Fig. S1): both candidate BQs and DPQs were phenotyped on both the early and late traits, with a maternal and paternal generation interval of 2 years; each BQ produced 18 candidate BQs and 18 candidate DPQs per generation; and the yearly mortality was of 25% (Fig. 1). The last queens were born in year 30, after 10 generations of selection following the Base breeding scheme.

#### Alternative breeding scheme

In parallel and starting from the same Initialization outcomes, in Alt, BQs born in year 10 were selected in year 11 at 1 year old. All subsequent BQs were also selected at 1 year old. As a consequence, phenotyping of candidate BQs was limited to the early trait only (Fig. 1). In contrast, candidate DPQ remained selected at 2 years old after complete phenotyping (i.e., phenotyping of both traits), as in Base and the Initialization. As in Base, BQs were selected based on EBVs to produce offspring BQs and DPQs. In this transition year 11 from the Initialization to the Alt scheme, the offspring BQs were mated by the same pool of selected DPQs (born in year 8) as BQs born in year 10, due to the lack of DPQs born in year 9 in the Initialization phase. After year 11, BQs were always mated by 2-year-old DPQs. Due to earlier selection in Alt, BQs faced only one annual mortality event before selection, increasing the number of candidates to selection from 243 to 324. As a result, the selection rate for BQs decreased from 9.9% (Base) to 7.4% (Alt), increasing the selection intensity from 1.76 in Base to 1.89 in Alt. In contrast, DPQs always had the same selection rate of 9.9% in all simulation phases. The last queens were born in year 30 as in Base. However, due to the halved dam generation interval, at the end of the simulation, 20 dam generations were produced in Alt instead of 10 in Base.

#### Genetic connection across apiaries

Newborn BQs and DPQs were allocated to 18 apiaries in two independent draws for each queen type before the first mortality event. The resulting genetic connections between apiaries were as follows (we indicate average remaining numbers after mortality in brackets): each group of 18 sister queens was split on 3 apiaries, and each apiary tested 4 sister groups, each comprising 6 (4.5) queens. Each apiary thus tested 24 (18) BQs and 24 (18) DPQs. This resulted, on average, in 36 surviving colonies for phenotyping per apiary and test year. This represents a rather large number of colonies per test apiary, however still in the range reported, e.g., by Kistler et al. (2024a) in a real breeding plan.

#### Additional scenarios with increased selection percentages

In addition to the focal set of simulations, additional ones were run with an increased number of parents selected to produce the new generation (36 selected BQs and 36 selected DPQs). The total candidate population size and the number of apiaries were kept constant. Therefore, the selected proportions were increased on both dam and sire paths. Sister-group sizes were reduced from 18 to 12 survived potential queens on each path. Everything else remained the same. Results are only presented in the Tables S7–S10.

### Genetic parameter sets and apiary effects

The residual and additive genetic variances in the base population were the same for both the early and the late trait.

The queen effect variance (${\sigma }_{{A}^{{Q}}}^{2}$) was half that of the worker effect (${\sigma }_{{A}^{{W}}}^{2}$) and a third of the residual effect (${\sigma }_{\varepsilon }^{2}$). These ratios of variances are in the range of real dataset estimates for most traits of interest, such as honey yield, royal jelly yield, swarming drive, and hygienic behavior (Brascamp et al. 2016, 2018; Hoppe et al. 2020; Basso et al. 2024).

Results will focus on simulations using a null genetic correlation between worker and queen effects (${r}_{W{,}Q}$). However, as often a negative ${r}_{{W}{,}{Q}}$ is estimated in real datasets, a complete set of results for ${r}_{{W}{,}{Q}}=-0.5$ is also given in Supplementary Material.

Lastly, the genetic correlation between the early and the late (${r}_{T1,T2}$) trait was set to vary from [−0.6, +0.6] by 0.3 intervals.

As both traits had the same genetic parameters, the resulting (co)variance matrix for both effects and traits can be obtained as the following Kronecker product:

$$\left[\begin{array}{cc}1 & {r}_{T1,T2}\\ {r}_{T1,T2} & 1\end{array}\right]\otimes \left[\begin{array}{cc}{\sigma }_{{A}^{{W}}}^{2} & {\sigma }_{{{A}}^{{W}{Q}}}\\ {\sigma }_{{{A}}^{{W}{Q}}} & {\sigma }_{{A}^{{Q}}}^{2}\end{array}\right]$$

with ${\sigma }_{{{A}}^{{W}{Q}}}$ the genetic covariance between worker and queen effects.

Scenarios were defined by the combination of all breeding goals and all ${r}_{T1,T2}$ values.

Simulated apiary effects for each year were drawn independently from a normal distribution. For the early and late trait, the variances of these apiary effects were set to represent approximately 1/3 and 2/3 of the phenotypic variance, respectively. These values are as estimated by Kistler et al. (2024a), taking hygienic behavior and honey yield as examples of possible early and late traits.

### Estimation of genetic parameters and breeding values

Before each selection step in both Base and Alt, genetic parameters were estimated using a ReML approach applied to a 2-trait mixed linear model with worker and queen genetic effects (colony model) (Chevalet and Cornuet 1982; Bienefeld et al. 2007) and a fixed environmental effect for the apiary-by-year effects. Estimation of genetic parameters during selection was done to account for the lack of precision of estimates, which affects selection accuracy in practice. Since the amount and structure of data available per trait differed between Base and Alt, this approach ensured that these differences were considered in the selection outcomes and thus in the comparison of Base and Alt scenarios.

All available phenotypes of present and past selection candidates at the moment of selection were used to run the genetic evaluation, regardless of the breeding goal, and thus included in the performance file the phenotypes of colonies in the Initialization phase. This amounted to a minimum of 3264 records on each trait at the first estimation in Base (in year 12), and 2616 records on the late trait and 2940 on the early trait at the first estimation in Alt (in year 11). Note that in Alt, the early trait of 1-year-old DPQs was not included in the performance file, appearing only the following year together with the late trait, when these DPQs were 2 years old and became candidates to selection. In the last year, the performance file contained 9096 records for each trait in Base, while in Alt, it contained 8772 for the late trait and 15,252 for the early trait.

The pedigree was completed assuming that each open-mated queen (BQs in years 1 and 2, and all DPQs) was mated to drones produced by a different pseudo sire composed of 100 unknown and unrelated dummy DPQs (see Kistler et al. 2024b). Later BQs, which were inseminated, had the single DPQ producing the drones mating them assigned as mate. This pedigree was used to obtain the inversed relationship matrix (${{\bf{A}}}^{-1}$), containing a line and column for each dummy open mating pseudo sire, queen, and its worker group, following Brascamp and Bijma (2014, 2019). Two pieces of R software to produce ${{\bf{A}}}^{-1}$ are available (Brascamp and Bijma 2019): pedigree-20.R and AMD-AINV-20.R. The first converts the entry pedigree to one with a line for every queen, sire, and worker group, while storing parameters in additional files. The second uses those inputs to build **A** and its inverse. Both have been wrapped in R functions and integrated into the simulation software, which has been made publicly available (see section “Data availability”). The simulation code makes use of the R packages tidyr and dplyr (Wickham et al. 2019), data.table (Barrett et al. 2023), and mvtnorm (Genz and Bretz 2009).

The performance file and ${{\bf{A}}}^{-1}$ were used as input for AIREMLF90 (ver. 1.149) of the BLUPF90 package (Misztal et al. 2002) to solve the BLUP equations. Starting parameter values for the convergence algorithm were set as, respectively, 0.9 and 1.1 times the true value for genetic and for residual variances. The convergence criterion was set to ${1e}^{-11}$. For each scenario, 50 to 55 independent replicate simulations were run. If convergence failed in either Base or Alt at any estimation, simulation stopped, recorded the fail, and a new replicate was run.

### Summary statistics

The genetic gain and inbreeding coefficients in the Alternative or Base breeding schemes were expressed compared to their levels for queens born in year 10 (end of Initialization).

To compare Alt and Base, relative differences were calculated as:

$${\rm{relative\; difference}}=\frac{{\rm{value\; in\; Alt}}-{\rm{value\; in\; Base}}}{\left|{\rm{value\; in\; Base}}\right|}$$

Results will show the relative differences calculated within repetitions and averaged across repetitions.

Alt’s empirical probability of bringing more genetic gain for the breeding goal than Base ($\hat{p}$) was estimated in each scenario as the percentage of replicated simulations in which Alt achieved strictly higher gains than Base. The standard error of this binomial variable was estimated as:

${\rm{S}}{\rm{E}}=\sqrt{\frac{\hat{{\rm{p}}}(1-\hat{{\rm{p}}})}{{\rm{n}}}}$, with *n* the num of replicated simulations.

## Results

Across all scenarios, convergence at each ReML estimation in both Base and Alt was reached for at least 80% of repetitions. Parameters most impeding convergence were a null ${r}_{{W}{,}{Q}}$ and strong $\left|{r}_{T1,T2}\right|$ values.

**Fig. 2:**
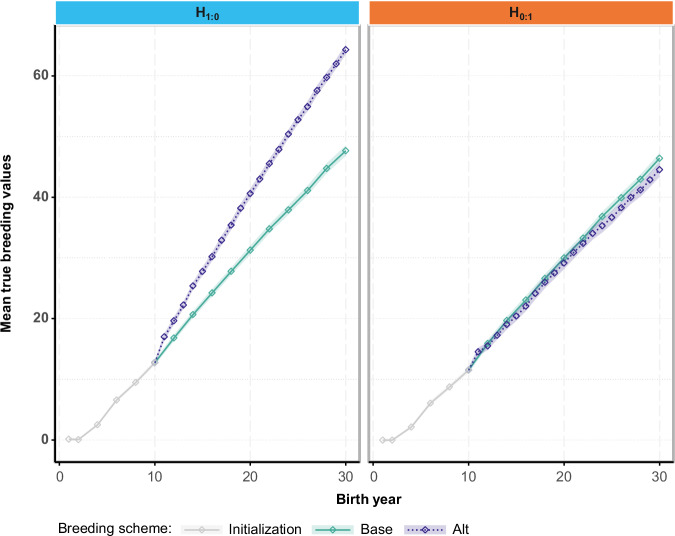
Mean genetic values of potential dams across years of birth for selection on either the early (*H*_1:0_) or late trait (*H*_0:1_), for scenarios with a null genetic correlation between worker and queen effects within a trait. *H*_1:0_: breeding goal comprising only the early trait. *H*_0:1_: breeding goal comprising only the late trait. Initialization: this phase lasted from year 1 to year 10 included, during which the population is closed and sufficient information is accumulated for the first ReML parameter estimation that will follow. In parallel and starting from the same Initialization outcomes, the population was then subjected to EBV selection using estimated parameters while following the Base or Alt breeding scheme. Base: reference breeding scheme with complete phenotyping and a 2-years generation interval on both the dam and the sire path. Alt: accelerated breeding scheme in which only the early trait is phenotyped on potential dams, while potential sires are also phenotyped on the late trait. This partial phenotyping of the dams enables halving the dam generation interval to 1 year.

The increase in breeding values (Fig. 2) and inbreeding coefficients (data not shown) was near linear after the Initialization phase, so that results at mid-term (after 10 years of selection following the Base or Alt breeding scheme) can be approximately inferred from results at long-term (20 years). Results will focus on the long-term outcomes.

### Genetic gain

#### Relative difference between Alt and Base in long-term genetic gains

**Fig. 3:**
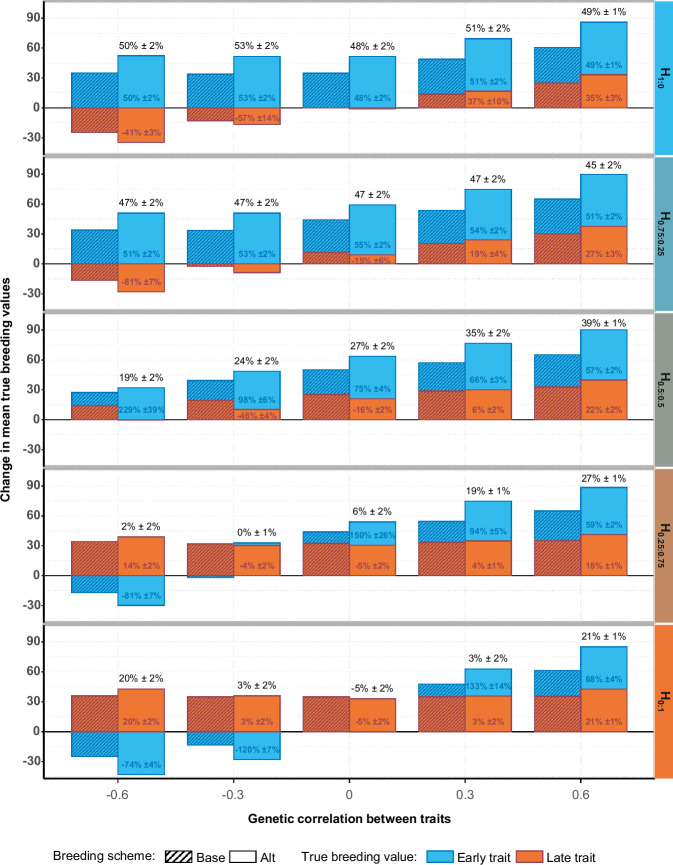
Change in mean true breeding values of selection candidates since the end of Initialization and their relative difference between Alt and Base for the breeding goal and per trait, after 20 years, for scenarios with a null genetic correlation between worker and queen effects within a trait. H: breeding goal. The first number in the subscript refers to the weight on the early trait and the second number to the weight on the late trait. Base: reference breeding scheme with complete phenotyping and a 2-years generation interval on both the dam and the sire path. Alt: accelerated breeding scheme in which only the early trait is phenotyped on potential dams, while potential sires are also phenotyped on the late trait. Partial phenotyping of the dams enables halving the dam generation interval to 1 year. Breeding values are calculated as the change since the end of the Initialization phase and after 20 years of selection following the Base or Alt breeding scheme. Text values shown represent the relative differences between the outcomes of Alt and Base, calculated within repetitions and averaged across repetitions. These averaged relative differences were calculated for each trait (within bars) or for the breeding goal (above the stacked bars).

Across all scenarios, Alt outperformed or equaled the average genetic gain obtained in Base except for the scenario focusing only on the late trait (*H*_0:1_) when ${r}_{T1,T2}$ was null (Fig. 2, right pane). The averaged relative difference between Alt and Base in the genetic gain for the breeding goal went from −5% to +53% (values on top of the stacked bars in Fig. 3 and Table S1). Alt’s superiority in genetic gain was highest when the breeding goal focused only on the early trait (Fig. 2, left pane, Fig. 3 and Table S1), and it diminished as more weight was placed on the late trait. Alt brought similar relative gains compared to Base, regardless of ${r}_{T1,T2}$, when the breeding goal focused on the early trait. This changed when more emphasis was placed on the late trait (*H*_0.5:0.5_ and H_0.25:0.75_), where Alt brought greater gains for positive ${r}_{T1,T2}$ values than for null or negative values. In the extreme case where all the weight was on the late trait (*H*_0:1_), genetic gains in Alt relative to Base were equal for equal $\left|{\,r}_{T1,T2}\,\right|$ values, with larger $\left|{\,r}_{T1,T2}\,\right|$ being more beneficial to Alt.

When worker and queen effects were negatively correlated (see Fig. S3 and Table S2) genetic gain decreased in both breeding schemes, but more so in Alt than in Base for breeding goals with equal or more weight on the late trait (*H*_0.5:0.5_, *H*_0.25:0.75_, and *H*_0:1_).

Figure 3 also illustrates that the contribution of the two traits to the genetic gain for the breeding goal varies greatly between the two breeding schemes across scenarios. For example, for *H*_0.5:0.5_ and a null *r*_*T*1*T*2_, Alt outperformed by +27% Base for the gain on the breeding goal only due to its superior progress on the early trait (+75%), while it performed less well than Base for the late trait (−16%).

Lastly, the average relative difference between Alt and Base came from different gains in worker and queen effects across the breeding goals (Figs. S2 and S3). When the breeding goal emphasized the early trait, the superior genetic gain in Alt came from a mere balanced superiority in responses for the worker and queen effects in comparison to those observed with Base. However, as the breeding goal put more weight on the late trait, Alt’s superiority was achieved through higher gains for queen effects than for worker effects relative to Base. This was due to Alt achieving less gains on worker effects as the breeding goal put more weight on the late trait, rather than to Alt increasing its gains on queen effects. For example, for *H*_0.25:0.75_ and ${r}_{T1,T2}=0$, Alt brought 7% less genetic gain on worker effects, but this was more than compensated by a +36% relative increase on queen effects, resulting in a +6% gain on the breeding goal compared to Base (Fig. S2).

#### Selection accuracy in the Alternative compared to the Base breeding scheme

**Fig. 4:**
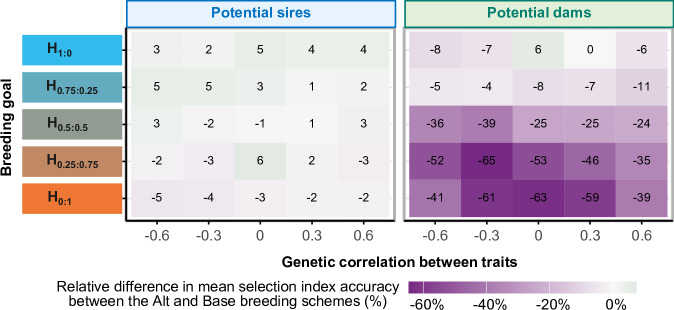
Relative difference in the mean index accuracy between the Alternative and Base breeding schemes for potential sires and potential dams, for scenarios with a null genetic correlation between worker and queen effects within a trait. Base: reference breeding scheme will complete phenotyping and a 2-years generation interval on both the dam and the sire path. Alt: accelerated breeding scheme in which only the early trait is phenotyped on potential dams, while potential sires are also phenotyped on the late trait. This partial phenotyping of the dams enables halving the dam generation interval to 1 year. The selection index accuracy was calculated as the correlation coefficient between the true and the estimated selection index. The relative difference in the mean selection index accuracy in the Alternative (Alt) and that in the Base breeding schemes was calculated within simulation runs and averaged across. Values shown are for the last year of simulation (year 30), expressed in precentages. Standard errors of relative differences in mean selection accuracy varied from 2% to 5%. The mean accuracy in Base, relative to which the differences for the accuracies in Alt are shown, varied from 0.46 ± 0.01 to 0.62 ± 0.01 depending on the scenario.

Figure 4 shows the relative difference in the mean accuracy between Alt and Base for potential sires and potential dams, for all scenarios with a null genetic correlation between queen and worker effects. Regardless of the breeding goal, the sire index accuracies were roughly similar in both breeding schemes, thus when phenotypes for both traits were available directly on candidates in Alt as in Base (see Fig. 4, left panel). On the opposite, mean relative differences in dam index accuracies varied widely from +6% to −63% in Alt compared to Base, depending on the breeding goal and the between-trait genetic correlation (see Fig. 4, right panel). The stronger reduction in accuracy arose for breeding goals emphasizing the late trait, which was not phenotyped on candidate dams in Alt. The raw values of index accuracy used as the base to which the relative differences are shown in Fig. 4 can be found in Fig. S4.

#### Probability of the Alternative breeding scheme to outperform the Base breeding scheme

Table 1 shows the empirical probability of Alt to outperform Base in terms of genetic gain for the breeding goal and for each trait.

**Table 1:** Empirical probability of the Alternative breeding scheme to bring strictly more genetic gain than the Base one after 20 years of selection.

Breeding goal (*H*)	*r*_*T*1,*T*2_	Empirical probability of the Alt breeding scheme to bring more genetic gain than the Base one					
		For *H*		For *T*1		For *T*2	
		Mean	SE	Mean	SE	Mean	SE
*H*_1:0_	0	1.00	NE	1.00	NE	0.48	0.07
	−0.6	1.00	NE	1.00	NE	0.02	0.02
	0.6	1.00	NE	1.00	NE	0.98	0.02
*H*_0.75:0.25_	0	1.00	NE	1.00	NE	0.32	0.06
	−0.6	1.00	NE	1.00	NE	0.00	NE
	0.6	1.00	NE	1.00	NE	0.98	0.02
*H*_0.5:0.5_	0	0.98	0.02	1.00	NE	0.14	0.05
	−0.6	0.90	0.04	1.00	NE	0.00	NE
	0.6	1.00	NE	1.00	NE	0.98	0.02
*H*_0.25:0.75_	0	0.68	0.07	0.94	0.03	0.28	0.06
	−0.6	0.62	0.07	0.02	0.02	0.86	0.05
	0.6	1.00	Ne	1.00	NE	1.00	NE
*H*_0:1_	0	0.26	0.06	0.50	0.07	0.26	0.06
	−0.6	0.94	0.03	0.00	NE	0.94	0.03
	0.6	0.98	0.02	1.00	NE	0.98	0.02

*NE* non-estimable.

Base: reference breeding scheme with complete phenotyping and a 2-years generation interval on both the dam and the sire path. Alt: accelerated breeding scheme in which only the early trait is phenotyped on potential dams, while potential sires are also phenotyped on the late trait. This partial phenotyping of the dams enables halving the dam generation interval to 1 year.

When all or most the weight in the breeding goal was on the early trait, the empirical probability of Alt to bring more genetic gain for the breeding goal than Base was 1, and at least 0.9 when weights on each trait in the breeding goal were equal. Shifting the emphasis to the late trait, this probability decreased to 0.68 for *H*_0.25:0.75_ and a null ${r}_{T1,T2}$. Still for a null ${r}_{T1,T2}$ but when all the weight was on the late trait, the probability for Alt to bring more genetic gain on the breeding goal than Base was only 0.26, this scenario being the worst for Alt.

### Inbreeding

#### Long-term inbreeding coefficients

**Fig. 5:**
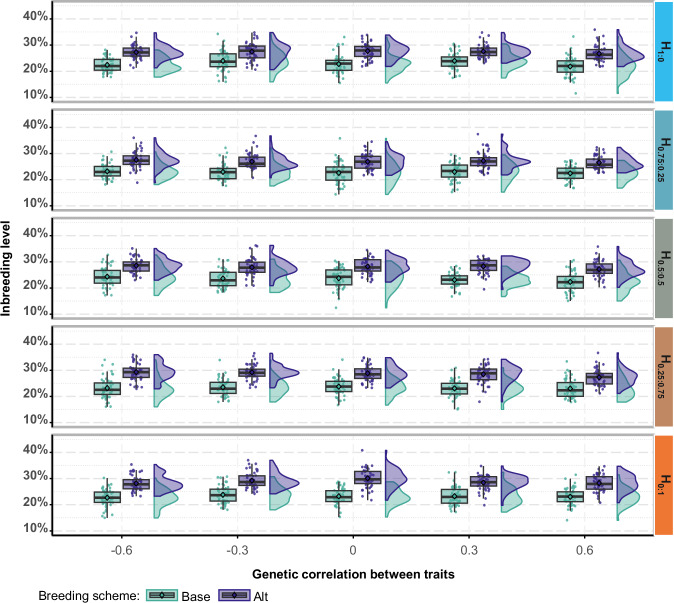
Change in mean inbreeding coefficients since the end of initialization in the Base and Alt breeding scheme, for all scenarios with a null genetic correlation between worker and queen effects within a trait. H: breeding goal. The first number in the subscript refers to the weight on the early trait and the second number the weight on the late trait. Base: reference breeding scheme will complete phenotyping and a 2-years generation interval on both the dam and the sire path. Alt: accelerated breeding scheme in which only the early trait is phenotyped on potential dams, while potential sires are also phenotyped on the late trait. Partial phenotyping of the dams enables halving the dam generation interval to 1 year. The curves represent density curves based on the outcomes of each replicate for a given simulation scenario.

The increases in mean inbreeding coefficients reached on the long term were similar across all scenarios for each breeding scheme (Table S3). Individual results could vary considerably between replicates (Fig. 5). They averaged 23% in Base and 28% in Alt for ${r}_{{W}{,}{Q}}=0$ (Table S3). In Base, it reached its maximum at 24% for *H*_0.5:0.5_ with the strongest negative ${r}_{T1,T2}$ (Table S3). The relative increases were higher in Alt by 17 ± 3% to 31 ± 3% compared to Base (+23% on average, representing increases from +0.04 to +0.07), with larger values for breeding goals emphasizing the late trait (Table S3). The increase in mean inbreeding coefficients in Alt since the end of Initialization reached a maximum value of 30% for *H*_0:1_ and ${r}_{T1,T2}=0$ (Table 2).

**Table 2:** Increase in mean inbreeding coefficients (%) after the end of Initialization and its relative difference between Base and Alt for the two single-trait breeding goals and scenarios with a null genetic correlation between worker and queen effects within a trait.

Breeding goal (*H*)	Added mean inbreeding coefficient (%)				Relative difference between both breeding schemes (%)	
	Base breeding scheme		Alternative breeding scheme			
	Mean	SE	Mean	SE	Mean	SE
*H*_1:0_	22.75	0.48	27.69	0.41	24.31	3.17
*H*_0:1_	23.23	0.43	30.11	0.50	31.23	2.65

Base: reference breeding scheme with complete phenotyping and a 2-years generation interval on both the dam and the sire path. Alt: accelerated breeding scheme in which only the early trait is phenotyped on potential dams, while potential sires are also phenotyped on the late trait. This partial phenotyping of the dams enables halving the dam generation interval to 1 year.

Results for the same scenarios but with ${r}_{{W}{,}{Q}}=-0.5$ were very similar, with the same or a maximum of +2% of added inbreeding coefficients (Table S4).

## Discussion

We used simulation to quantify the impact of reducing the classical 2-years dam generation interval in honeybee breeding schemes to 1 year. This shortening in the dam’s generation interval implied a partial phenotyping limited to some early recorded traits. The sire generation interval remained at 2 years with phenotyping of both early and late traits. The effect of various breeding goals was studied, emphasizing either an early or a late trait or equally weighting both. Different genetic correlations between both traits were also considered.

Annual genetic gain for the breeding goal was generally greater with the shorter generation interval strategy than with the base scenario with the longer generation interval strategy. This greater gain was achieved more through the gain in the early trait compared to the gain in the late trait. It came, however, at the cost of a relative increase in mean inbreeding coefficients of roughly +20% to +30% after 20 years of selection in Alt compared to Base (representing an increase in mean inbreeding coefficients of about +0.04 to +0.07).

### Major impact of the dam index accuracy on the differences in relative gain between Alt and Base across breeding goals

Regardless of the breeding goal, response to selection was favored in Alt due to a 25% reduction in the average generation interval and a $7.5 \%$ increase in dam selection intensity, as earlier dam selection avoided a second random mortality event. Still regardless of the breeding goal, selection index accuracies were roughly similar for sires in both the Alt and Base breeding schemes. The differences in genetic gain between the two schemes across breeding goals can largely be attributed to the relative difference in dam selection accuracies (Fig. 4). The more the breeding goal emphasized the late trait, which was not phenotyped on potential dams in Alt, the more the dam selection accuracy was reduced in Alt compared to Base. These differences across breeding goals were also strongly influenced by ${r}_{T1,T2}$. We analyzed dam index accuracies in Alt using selection-index theory (Hazel 1943) for a two-trait breeding goal with only one trait used as selection criterion to compare them with the empirical accuracies obtained by simulation (see File S2). Simulations confirmed the expected theoretical patterns, with both empirically and deterministically calculated accuracies reaching maxima and minima at the same ${r}_{T1,T2}$ values and the same breeding goals.

In our study, the early and late traits had identical genetic and residual variances. In reality, their heritabilities may differ, affecting the relative genetic gain between the Alt and Base breeding schemes. Based on general selection index theory, we expect that a higher heritability of the early trait would generally advantage Alt more than Base, while a lower heritability of the early trait would favor Base more. More specifically, for single-trait breeding goals and a null between-trait genetic correlation, the relative genetic gains between the two scenarios are expected to be independent of the early trait heritability. When the breeding goal is a mix of the two traits, then with a lower early trait heritability, Alt might be favored in less cases against Base.

While maintaining early dam selection, candidate dams could be phenotyped after selection to increase the accuracy in Alt for breeding goals emphasizing the late trait. By recording the late trait for older queens, this data would be integrated in the EBVs of the parents and, consequently, in the EBVs of the new candidate dams at the moment of selection. Doing so in simulation for *H*_0:1_ and null values for ${r}_{T1,T2}$ and ${r}_{{W}{Q}}$ increased the dam selection accuracy from 0.21 to 0.28, resulting in a 21% increase in genetic gain. Even in this least favorable scenario to Alt, more genetic gain could then be achieved using this parental information than with Base (+17% compared to −5% when these late-trait records on older candidate dams were absent, data not shown). If the phenotyping of late traits comes at a reduced cost, as arguably for total honey yield, later recording might thus be of interest to get a more efficient breeding scheme.

### Inbreeding rate per year or per generation

The Alt breeding scheme appeared to be a favorable strategy for genetic gain but unfavorable for inbreeding rate per year. The highest difference in inbreeding coefficients reached after 20 years between the two breeding schemes was observed for a null ${r}_{T1,T2}$ and when the breeding goal focused on the late trait, which was not phenotyped on candidate BQs. In the absence of phenotypes on candidates, using BLUP-EBVs, all sister-queens had similar EBVs for the target trait, corresponding to the mean of their parents’ EBVs (they still differed due to the fact that the between trait correlation was estimated and not fixed to 0). This probably resulted in the selection of BQs originating mostly from only the few best sister groups, increasing inbreeding more rapidly than when better discrimination within sister-groups could be made. Across scenarios, the 17% to 31% higher inbreeding coefficients in Alt was mainly due to Alt’s 33% increase in the average number of generations compared to Base during the same period, the inbreeding rate per generation being actually reduced in Alt by −2% ± 2% to −12% ± 2% per generation (−8% on average, Tables S5 and S6).

However, in both breeding schemes and for all breeding goals and between-traits correlations, inbreeding rates were above two times the common recommended maximum 1% rate (FAO 2013) per generation. It might be noticed, however, that breeding schemes in reality, especially in honeybees, probably do not remain genetically strictly closed for decades. However, maintaining the population closed and its size fixed, inbreeding rates could be lowered by relaxing the selection intensity, such as by selecting more breeding queens as in our additional simulations with 36 selected BQs and DPQs per generation (instead of 24, representing 33% less selected queens). This strategy effectively reduced inbreeding rates (near but slightly under the expected −33% reduction, Tables S7 and S8), while reducing genetic gains by only −5% on average across all scenarios whatever the ${r}_{{W}{Q}}$ value (Tables S9 and S10). In particular, genetic gain was at most only marginally reduced in Alt with 36 BQs, while the increased number of selected queens reduced mean inbreeding rates by 24% ± 2% to 33% ± 2% (Tables S7 and S8). Alt with 36 BQs thus led to at least almost as much genetic gain than Base with 24 BQs (−2% ± 2% for *H*_0:1_ and ${r}_{T1,T2}=0$) or significantly better (around +41% ± 2% for *H*_1:0_ whatever the ${r}_{T1,T2}$), while also bringing less long-term inbreeding coefficients (−5% for *H*_0:1_ and ${r}_{T1,T2}=0$, to −18% for *H*_1:0_ & ${r}_{T1,T2}=-0.6$). This shows that if a scheme similar to Alt is used in practice, maintaining a sufficient population size becomes more important to limit inbreeding rates per year due to the accelerated generation turnover. The relaxed intensity can come at practically no costs in genetic gain, which will benefit from the increased genetic variance remaining, while the reduced generation interval enables benefitting from the greater genetic gains per year. In practice, relaxing the selection intensity at a fixed breeding population size could add management costs, however. These added costs would be linked to rearing queens from more dams and inseminating with drones from more DPQs.

Another approach to relax selection intensity in order to maintain genetic diversity could have been to perform within-family selection (Kistler et al. 2021). However, an optimal way to compare Alt and Base for mean genetic gains at equal levels of inbreeding would have been to use Optimal Contribution Selection (Meuwissen 1997) to fix a predetermined inbreeding rate. Unfortunately, this method still has to be transposed to honeybees and their specificities.

### Inbreeding depression

Our study focused on the heritable component of phenotypic gain by addressing additive genetic gain. We accounted for the impact of inbreeding on additive genetic gain through its effect on reducing the Mendelian sampling variance (see Supplementary File S1), which limits the available additive genetic variability.

Beyond its direct effect on genetic variability, inbreeding can also affect genetic gain indirectly by increasing colony mortality due to inbreeding depression (ID), linked to deleterious recessive alleles. Added mortality would reduce selection intensity by decreasing the population size of selection candidates. As far as we know, the impact of inbreeding on colony survival remains largely unexplored, apart from brood losses caused by inbred workers with a higher probability of homozygosity at the sex locus (Woyke 1963). In haplo-diploid species like honeybees, recessive mutations creating lethal or highly deleterious alleles are fully expressed in haploid males before reproduction, allowing natural selection to purge these alleles (Crozier 1970). Despite this expected protective mechanism, honeybee populations can still suffer from ID (Moritz 1986; Bienefeld et al. 1989). ID could affect all kinds of traits, even traits not included in the breeding goal, and therefore impact the sustainability of the breeding program. Inbreeding of queens and of workers seems to affect colony traits differently, the main issues being observed with inbred workers. For instance, honey yield is significantly affected by workers’ inbreeding while no effect of queen’s inbreeding is observed by Bienefeld et al. (1989).

In principle, ID could be accounted for by discounting the estimated ID from the performance reached in each scenario. For example, Bienefeld et al. (1989) suggest a reduction of 140 g of honey production per 0.01 increase in inbreeding, amounting to −0.6% of their population’s mean per 0.01 increase in inbreeding. Applying this estimate to our scenario focusing on the late trait and with null correlations between traits and between genetic effects, where an average inbreeding coefficient of 0.23 was reached in Base vs. 0.30 in Alt (Table 2) at the final generation, performance levels would have been reduced from 34.89 to 20.96 in Base and from 33.02 to 14.96 in Alt (Tables S1 and S3).

We decided not to discount phenotypic gain for ID in our modeling, mainly due to the lack of population-specific estimates of ID in honeybees for all traits of interest. If desired, breeders can discount gains presented here with their expectation of ID, ideally estimated from their own population, using the results for gain and inbreeding presented here (Tables S1 and S3).

However, due to the difference in generation intervals, it is not trivial that the absolute inbreeding level at the final generation is the appropriate measure for ID. Indeed, in short-term selection experiments with high rates of inbreeding, e.g., in Drosophila (Latter and Robertson 1962), ID is typically related to the absolute inbreeding level. However, in ongoing breeding schemes, the focus should be on maintaining a sufficient effective population size so that the population is genetically sustainable in the long term (e.g., FAO 2013). Effective population size depends on the rate of inbreeding per generation, and the mechanisms counteracting inbreeding and ID, being mutation and purging of deleterious alleles by natural selection, also act on a per-generation basis (e.g., Ehiobu et al. 1989). Hence, this perspective would suggest to focus on the rate of inbreeding per generation, rather than the inbreeding level in the final generation.

### Reduced generation intervals and longevity

The concerning high and increasing colony losses in honeybees are partly due to issues in queen quality and longevity (Pettis et al. 2016; Amiri et al. 2017; Tang et al. 2023). We simulated colony mortality as a fully random event between 2 years. In reality, however, survival might not be entirely random, and longer generation intervals might reveal genetic differences in longevity. By shortening generation intervals, we cannot select on queen longevity which is a trait of major interest for beekeepers. An indirect and early selection criterion for queen longevity should be investigated, such as potentially queen fecundity, which is linked to queen longevity (Pettis et al. 2016; Amiri et al. 2017; Tang et al. 2023).

### Practical and economic considerations in implementing the Alternative over the Base breeding scheme

Adopting the Alt breeding scheme instead of the Base one would imply new practical and economic challenges. In particular, breeders would need, each year, to rear, fertilize, and manage twice as many candidate BQs and DPQs, doubling overall colony management costs. In addition, approximately twice as many colonies would need to be phenotyped for the early trait, while the total number of colonies assessed for the late trait would remain unchanged. The additional phenotyping costs in Alt would thus depend on the relative costs for phenotyping the early vs the late trait.

Furthermore, to have the most straight-forward comparison, the only difference between Alt and Base was the reduced dam generation interval. In consequence, in Alt, potential dams and sires were bred every year, as opposed to every even year in Base. In reality, breeders using a scheme similar to Base would have probably split their population in two, to breed new candidates in one subpopulation and phenotype the other every year, enabling them to increase the total population size and to cross-breed excelling individuals from one subpopulation with the other (Uzunov et al. 2022). This would likely benefit genetic gain and inbreeding.

### Shortening of the generation interval at no expense of phenotyping late traits

Breeders could potentially benefit from the accelerated response to selection obtained by shortening the dam generation interval without sacrificing the recording of late traits before dam selection. In a first approach, this could be achieved by delaying the reproduction period until after the recording of late traits. In temperate climates, a major challenge in this approach is to ensure that colonies rear enough drones late in the beekeeping season, in late summer or autumn. This may be feasible in certain areas, e.g. those with a Mediterranean climate and adequate floral resources during that period, depending on specific beekeeping practices, colony management, and environmental conditions.

Alternatively, a second approach could be to delay reproduction to after the measurement of late traits, but instead of relying on late-drone production which might be unreliable, semen could be collected earlier when drones are readily available, and stored for a few months (van Praagh et al. 2014; Hopkins et al. 2017). The semen could thus be used later, after dam selection on late traits, to inseminate the new candidates for selection.

Note that if the first approach can be used, i.e. delaying drone production, then shortening the generation interval without sacrificing selection on late traits could also be applied to sires, leading to a 1-year generation interval while maintaining complete phenotyping on both maternal and paternal paths.

In the future, when emerging conventional bee breeding schemes will have reached a sufficient maturity, genomic selection (Bernstein et al. 2021, 2023) could be a promising alternative to early select late traits if a cost-efficient genotyping strategy is developed for bees.

## Conclusion

Halving the dam generation interval to 1 year at the expense of phenotyping only the early trait on potential dams generally resulted in an increased genetic gain, up to +50% after 20 years of selection depending on the breeding goal. The highest increases were observed when the breeding goal focused primarily on the early trait or when the genetic correlation between the early and late traits was positive. In contrast, the accelerated breeding scheme achieved slightly lower genetic gain than the Base one when the breeding goal focused only on the late trait and when it was not correlated with the early recorded trait. Although inbreeding rates per generation were slightly reduced in the accelerated breeding scheme, inbreeding coefficients were still higher due to the increase in the number of generations obtained during the 20 years of selection. Shortening the generation interval should therefore be accompanied by strategies to limit long-term inbreeding. For example, when accelerating the breeding scheme, an effective strategy to limit inbreeding is to increase the breeding nucleus size by relaxing selection intensity. Doing so can substantially reduce inbreeding rates while still retain most of the genetic gain benefits compared to not shortening the average generation interval.

## Supplementary information

Supplementary material

## Data Availability

The R simulation code used in the current study has been made publicly available at the simu_bees GitHub repository http://github.com/Tristan-Kistler/simu_bees in the folder ‘partial_pheno_short_gen_interval_study’ and archived on Software Heritage, with the SWHID: swh:1:dir:4f183d1233df180713db7ef3395cbbb07a4e01f0;origin=https://github.com/Tristan-Kistler/simu_bees;visit=swh:1:snp:7b9d55a2a2a84e37152a47143857d58a37eb2c82;anchor=swh:1:rev:afed68dfd1d6a0691a21f99479dd2fc9e28de036;path=/partial_pheno_short_gen_interval_study/. Further developments will also be available via the GitHub repository. The R version used to run the above code was 3.5.0 (2018-04-23), but the code should be compatible with versions from 3.6.2 to 4.2.1.
