# Supplementary material for: How partial phenotyping to reduce generation intervals can help to increase annual genetic gain in selected honeybee populations
Source: Heredity (Edinb). 2025 May 22;134(7):396–407. doi: 10.1038/s41437-025-00768-8 (PMC12217302; doi:10.1038/s41437-025-00768-8)
Supplement: Supplementary file 1 — Supplementary material [file 41437_2025_768_MOESM1_ESM.pdf]

Supplementary files

File S1

Infinitesimal model applied to the honeybee for queens, drones, and worker groups Generation of base individuals:

$\mathbf{a}^{\mathbf{base\ BQs}} \sim \boldsymbol{N}(\mathbf{0}, \boldsymbol{\Sigma}^2_{\mathbf{a}})$: Breeding queens (BQ) from the base population had their additive genetic values **(a)** drawn from a bivariate normal distribution centered on **0** and with (co)variance matrix of both effects and traits $\boldsymbol{\Sigma}^2_{\mathbf{a}}$.

$\mathbf{a}^{\mathbf{base\ Ds}} \sim \boldsymbol{N}(\mathbf{0}, \tfrac{1}{2}\boldsymbol{\Sigma}^2_{\mathbf{a}})$: Base drones, which are haploid, had their additive genetic values made to (co)vary half as much as that of BQs.

After mating, queens produced offspring worker groups by:

$$\overline{\mathbf{a}^{\mathbf{W}}} = \frac{1}{2} \cdot \mathbf{a}^{\mathbf{Q}} + \overline{\mathbf{a}^{\mathbf{Ds}}}$$

where the additive genetic values of a worker group ($\overline{\mathbf{a}^{\mathbf{W}}}$) equaled the sum of half the additive genetic values of its BQ and the average additive genetic values of the drones that mated the BQ.

Colonies' phenotypes were obtained using:

$$\mathrm{y} = \mathrm{a}_{\mathrm{q}}^{\mathrm{Q}} + \frac{1}{2}\mathrm{a}_{\mathrm{w}}^{\mathrm{Q}} + \overline{\mathrm{a}_{\mathrm{w}}^{\mathrm{Ds}}} + \mathrm{E} + \varepsilon$$

where the performance (y) of a colony equaled the sum of the queen genetic effect expressed by the queen $\left(\mathrm{a}_{\mathrm{q}}^{\mathrm{Q}}\right)$, half the worker genetic effect of the queen $\left(\frac{1}{2}\mathrm{a}_{\mathrm{w}}^{\mathrm{Q}}\right)$, the average worker genetic effect of the drones that mated the queen, a non-heritable apiary by year effect (E), and a random residual (ε) modeling other non-additive genetic effects .

Offspring queens inherited their additive genetic values following:

$$\mathbf{a}^{\mathbf{Q}} = \frac{1}{2} \cdot \mathbf{a}^{\mathbf{BQ}} + \mathbf{a}^{\mathbf{D}} + \boldsymbol{\varphi}^{\mathbf{BQ}}$$

where their additive genetic values were the sum of half that of their BQ's, that of one of the drones that mated their BQ, and a mendelian sampling term ($\boldsymbol{\varphi}$) drawn from $\boldsymbol{N}(\mathbf{0}, \frac{1}{4}\cdot(1\text{-F})\cdot\boldsymbol{\Sigma}^2\mathbf{a})$, where F is the inbreeding coefficient of the offspring's BQ.

Lastly, drones inherited their additive genetic values following:

$$\mathbf{a}^{\mathbf{D}} = \frac{1}{2}\mathbf{a}^{\mathbf{DPQ}} + \boldsymbol{\varphi}^{\mathbf{DPQ}} \quad (6)$$

where haploid drones' additive genetic values were inherited only from their drone-producing queen (DPQ).

Further details are given in Kistler et al. [20].

File S2

Optimal selection index based on a single selection criterion for a 2-trait breeding goal

The following derivations are useful to predict the breeding index accuracy of dam candidates (BQs) in the Alternative (Alt) breeding scheme. To simplify, we will assume phenotypic selection, although the simulations used EBV selection, and omit all honeybee specificities.

In Alt, BQs are only phenotyped on a single trait (T_1), but selected for their merit regarding a 2-trait breeding goal (H).

The coefficient of the optimal index (I) can be calculated as (Smith, 1936; Hazel, 1943) :

$$\mathbf{b} = \mathbf{P}^{-1}\mathbf{G}\mathbf{a}$$

With **b** the vector of index weights (or coefficients), $\mathbf{P}^{-1}$ the inversed phenotypic (co)variance matrix, **G** the (co)variance matrix of the predictors in the selection index and the true breeding values in **H**, and **a** the vector of the breeding goal weights.

We note:

$\sigma^2_{T_1}$ and h^2_1, respectively, the phenotypic variance and the heritability of the early trait,
σ_{u_1} and σ_{u_2} the genetic standard deviations of, respectively, the early and late trait,
σ_{T_1,u_1} the covariance between the phenotypic value of the early trait and its genetic value, σ_{T_1,u_2} the covariance between the phenotypic value of the early trait and the genetic value of the late trait,
a_1 and a_2 the weights attributed, respectively, to the early and late trait in the breeding goal,
σ_{u_1,u_2} the genetic covariance between the early and late traits,
$r_{T1,T2}$ the genetic correlation between the early and late trait.

Let us derive b_1, the index weight attributed to the early trait in the case of a single selection criterion for a two-trait breeding goal:

$$\begin{aligned}
b_1 &= \frac{1}{\sigma^2_{T_1}}\begin{bmatrix}\sigma_{T_1,u_1} & \sigma_{T_1,u_2}\end{bmatrix}\begin{bmatrix}a_1\\ a_2\end{bmatrix} \\
&= \frac{1}{\sigma^2_{T_1}}\begin{bmatrix}\sigma^2_{u_1} & \sigma_{u_1,u_2}\end{bmatrix}\begin{bmatrix}a_1\\ a_2\end{bmatrix} \\
&= \frac{1}{\sigma^2_{T_1}}\left(\sigma^2_{u_1}a_1 + \sigma_{u_1,u_2}a_2\right) \\
&= h^2_1 \cdot a_1 + \frac{\sigma_{u_1}\sigma_{u_2}}{\sigma^2_{T_1}} \cdot r_{T1,T2} \cdot a_2 \\
&= h^2_1 \cdot a_1 + h_1\frac{\sigma_{u_2}}{\sigma_{T_1}} \cdot r_{T1,T2} \cdot a_2 \\
b_1 &= h^2_1 \cdot a_1 + h_1 h_2 \cdot r_{T1,T2} \cdot \frac{\sigma_{T_2}}{\sigma_{T_1}} \cdot a_2 \qquad (1)
\end{aligned}$$

The selection index accuracy (ρ_I) is defined as:

$$\rho_I = \sqrt{\frac{\sigma_I^2}{\sigma_H^2}}$$

Let us derive σ_I^2 and σ_H^2, starting with σ_I^2:

$$\begin{aligned}
\sigma_I^2 &= V(b_1 \cdot T_1) \\
&= b_1^2 \cdot \sigma_{T_1}^2 \\
&= b_1^2 \cdot \frac{1}{h_1^2} \cdot \sigma_{u_1}^2 \\
&= \frac{1}{h_1^2} \cdot \sigma_{u_1}^2 \cdot \left[h_1^2 \cdot a_1 + h_1 h_2 \cdot r_{T1,T2} \cdot \frac{\sigma_{T_2}}{\sigma_{T_1}} \cdot a_2\right]^2 && \text{using (1)} \\
&= \frac{1}{h_1^2} \cdot \sigma_{u_1}^2 \cdot \left[h_1^4 \cdot a_1 + h_1^2 h_2^2 \cdot r_{T1,T2}^2 \cdot \frac{\sigma_{T_2}^2}{\sigma_{T_1}^2} \cdot a_2^2 + 2h_1^2 a_1 \cdot h_1 h_2 \cdot r_{T1,T2} \frac{\sigma_{T_2}}{\sigma_{T_1}} \cdot a_2\right] \\
&= a_1^2 \sigma_{u_1}^2 h_1^2 + \sigma_{u_1}^2 h_2^2 \cdot r_{T1,T2}^2 \cdot \frac{\sigma_{T_2}^2}{\sigma_{T_1}^2} \cdot a_2^2 + 2\sigma_{u_1}^2 a_1 \cdot h_1 h_2 \cdot r_{T1,T2} \frac{\sigma_{T_2}}{\sigma_{T_1}} \cdot a_2 \\
&= a_1^2 \sigma_{u_1}^2 h_1^2 + a_2^2 \cdot h_1^2 h_2^2 \cdot r_{T1,T2}^2 \cdot \sigma_{T_2}^2 + 2a_1 a_2 \cdot h_1^2 h_2 \cdot r_{T1,T2} \sigma_{T_2} \sigma_{u_1} \\
&= h_1^2 \left(a_1^2 \sigma_{u_1}^2 + a_2^2 h_2^2 \cdot r_{T1,T2}^2 \cdot \sigma_{T_2}^2 + 2a_1 a_2 \cdot h_2 \cdot r_{T1,T2} \sigma_{u_1} \sigma_{T_2}\right) \\
&= h_1^2 \left(a_1^2 \sigma_{u_1}^2 + a_2^2 \sigma_{u_2}^2 \cdot r_{T1,T2}^2 + 2a_1 a_2 \cdot \sigma_{u_1} \sigma_{u_2} \cdot r_{T1,T2}\right) \\
\sigma_I^2 &= h_1^2 \left(a_1 \sigma_{u_1} + a_2 \sigma_{u_2} r_{T1,T2}\right)^2 && (2)
\end{aligned}$$

Let us now derive σ_H^2:

$$\begin{aligned}
\sigma_H^2 &= V(a_1 \cdot u_1 + a_2 \cdot u_2) \\
&= a_1^2 \sigma_{u_1}^2 + a_2^2 \sigma_{u_2}^2 + 2a_1 a_2 \sigma_{u_1,u_2} \\
\sigma_H^2 &= a_1^2 \sigma_{u_1}^2 + a_2^2 \sigma_{u_2}^2 + 2a_1 a_2 \cdot \sigma_{u_1} \sigma_{u_2} \cdot r_{T1,T2} && (3)
\end{aligned}$$

Thus, the selection index accuracy can be calculated as:

$$\begin{aligned}
\frac{\sigma_I}{\sigma_H} &= \sqrt{\frac{h_1^2 \left(a_1 \sigma_{u_1} + a_2 \sigma_{u_2} r_{T1,T2}\right)^2}{a_1^2 \sigma_{u_1}^2 + a_2^2 \sigma_{u_2}^2 + 2a_1 a_2 \cdot \sigma_{u_1} \sigma_{u_2} \cdot r_{T1,T2}}} && \text{using (2) and (3)} \\
&= \frac{\left|h_1 \left(a_1 \sigma_{u_1} + a_2 \sigma_{u_2} r_{T1,T2}\right)\right|}{\sqrt{a_1^2 \sigma_{u_1}^2 + a_2^2 \sigma_{u_2}^2 + 2a_1 a_2 \cdot \sigma_{u_1} \sigma_{u_2} \cdot r_{T1,T2}}} && (4)
\end{aligned}$$

The heatmap hereafter shows on the left pane the empirical selection index accuracies for potential dams (BQs) in the Alternative breeding scheme, in which BQs are not phenotyped on the late trait, obtained by simulation. The right pane shows these values as calculated using equation (4), and considering $\sigma^2_{u_1} = \sigma^2_{u_2} = 10$, and $h^2_1 = 0.25$:

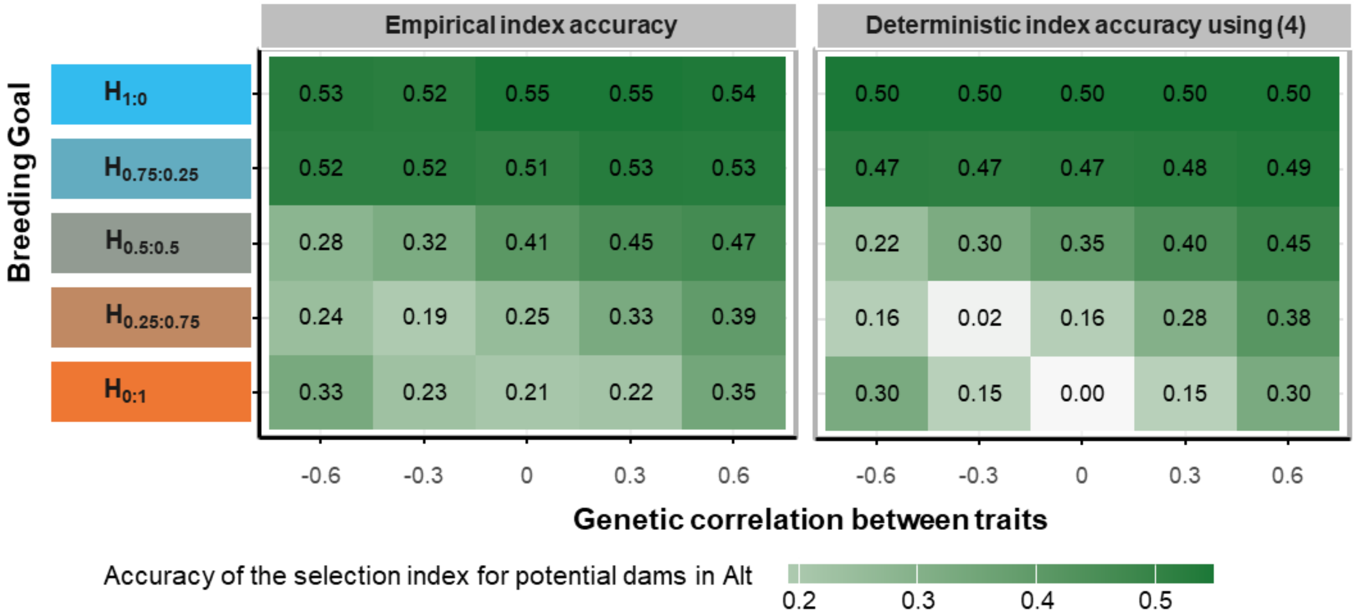

The empirical index accuracy was calculated as the correlation coefficient between estimated selection indices and aggregate true breeding values.

Because in the deterministic derivation, phenotypic selection was assumed out of simplicity, deterministic accuracy values were expected to differ from the empirical ones obtained by simulation. Nevertheless, we can see that both accuracy values follow similar patterns:

- When all the weight in the breeding goal is on the early trait ($\mathbf{H_{1:0}}$), measured on the selection candidate, both empirical and determinitic accuracies are constant or practically constant, irrespective of $r_{T1,T2}$.
- When weights in the breeding goal are the same for both traits ($\mathbf{H_{0.5:0.5}}$), both empirical and determinitic accuracies have their minima and maxima as does $r_{T1,T2}$.
- When all the weight in the breeding goal is on the late trait ($\mathbf{H_{1:0}}$), not measured on the selection candidates, both empirical and determinitic accuracies have their minima and maxima as does $|r_{T1,T2}|$.
- In the intermediary case with the two latter ones ($\mathbf{H_{0.25:0.75}}$), both empirical and determinitic accuracies have a minimum intermediary to the minimum of $r_{T1,T2}$ and $|r_{T1,T2}|$.

Supplementary figures

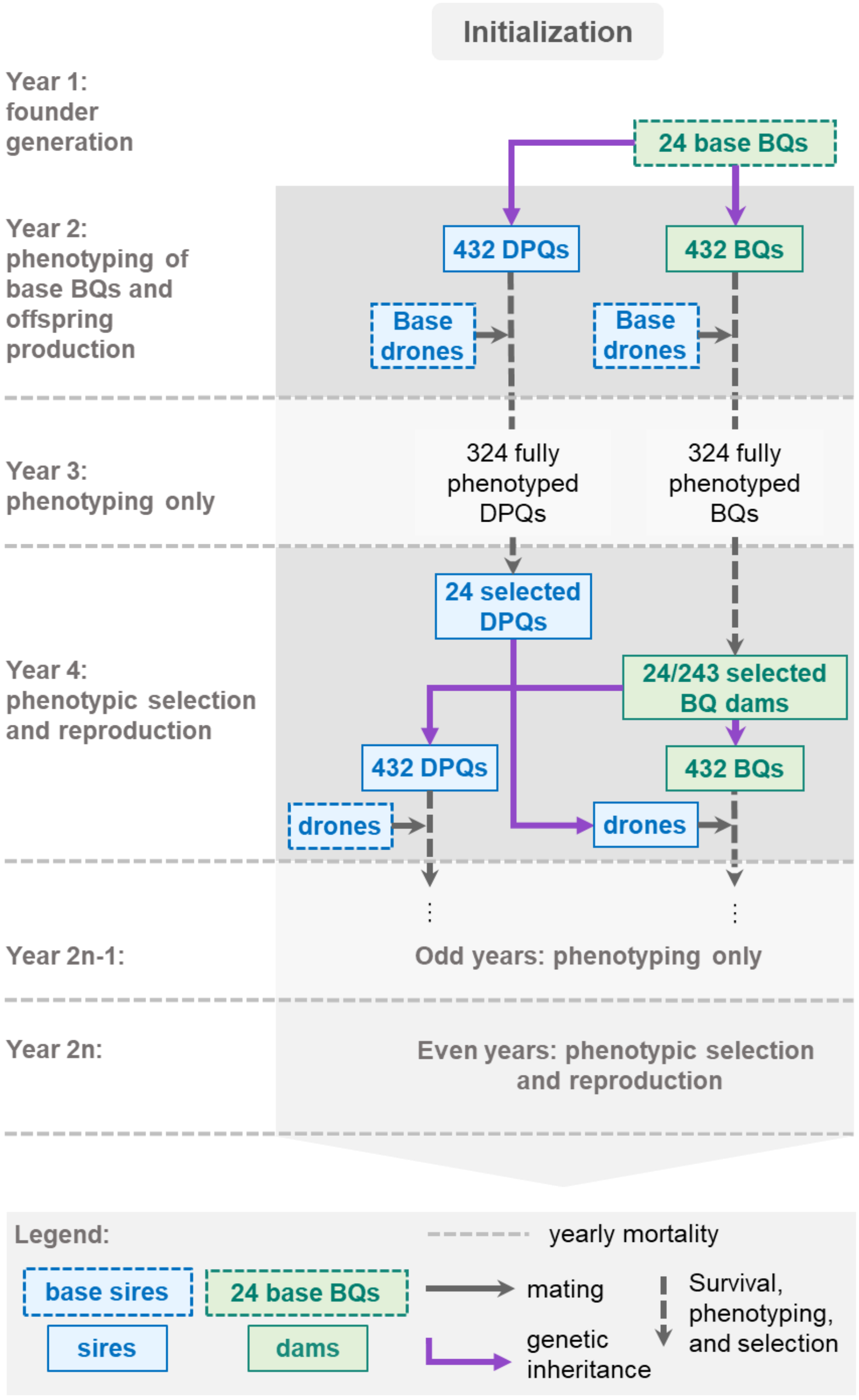

Fig. S1 Simulation process of the Initialization phase, with a 2-year generation interval and complete phenotyping on dam and sire paths

BQ: breeding queen.
DPQ: drone-producing queen.
BQ and DPQ refers to queens that were candidates for selection, and “selected BQ” or “selected DPQs” to selected dams and sires, respectively.
The Initialization phase lasted until year 10 included and served to establish a closed breeding population while accumulating sufficient pedigree and phenotypic records for subsequent REML variance component estimation. After Initialization, in year 11, the population followed two independent breeding schemes in parallel: (1) the Base scheme, which maintained the same structure as the Initialization phase but applied EBV-based selection, and (2) the Alternative scheme, which also used EBV-based selection but shortened the dam generation interval to one year, phenotyping only the early trait on potential dams.

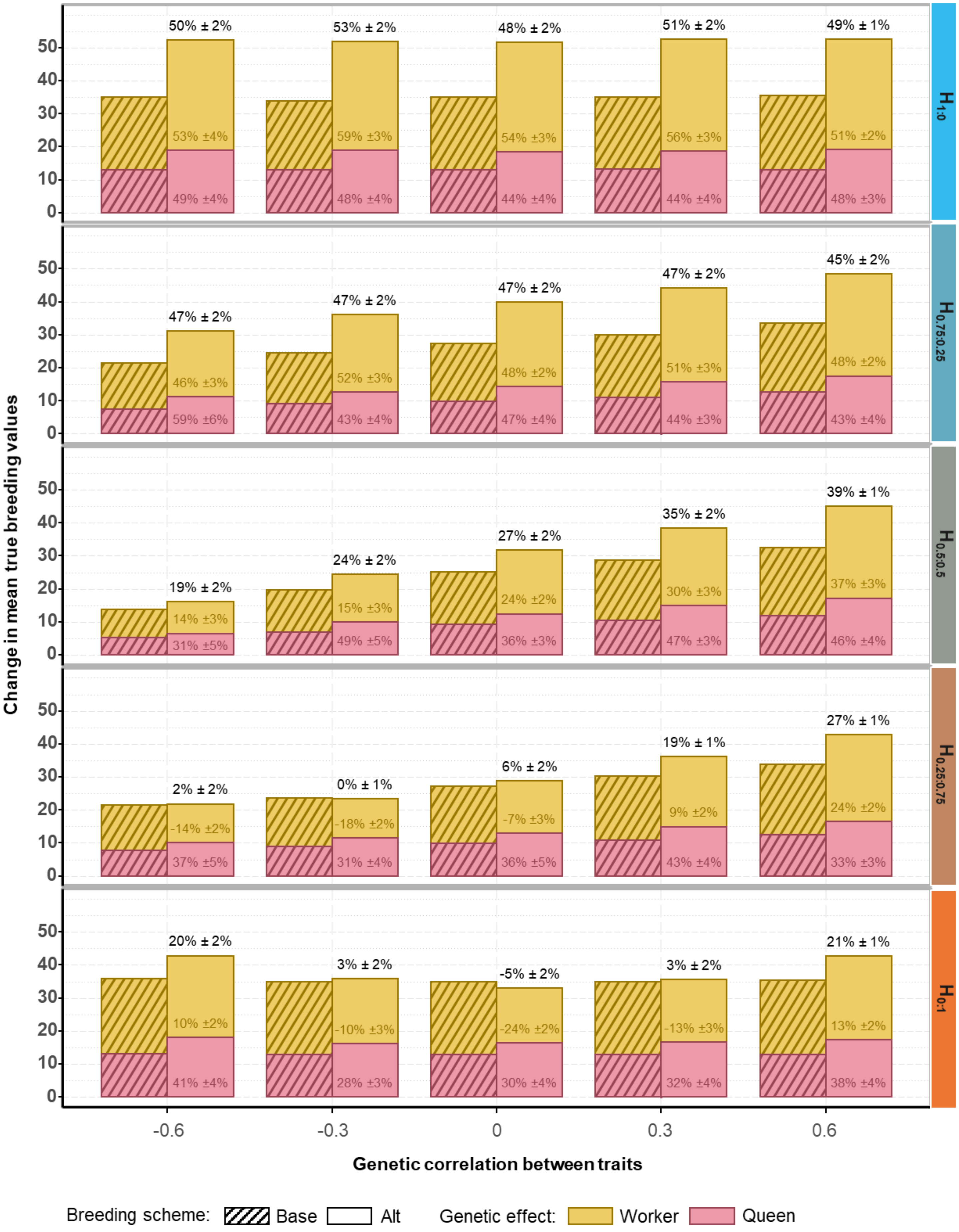

Fig. S2 Change in mean true breeding values since the end of Initialization and their relative difference between Alt and Base per effect and for the breeding goal after 20 years, for scenarios with a null genetic correlation between worker and queen effects within a trait.

H: breeding goal. The first number in the subscript refers to the weight on the early trait and the second number the weight on the late trait.
Base: reference breeding scheme will complete phenotyping and a 2-years generation interval on both the dam and the sire path.
Alt: accelerated breeding scheme in which only the early trait is phenotyped on potential dams, while potential sires are also phenotyped on the late trait. Partial phenotyping of the dams enables halving the dam generation interval to 1 year.
Breeding values are calculated as the change since the end of the Initialization phase and after 20 years of selection following the Base or Alt breeding scheme.
Text values shown represent the relative differences between the outcomes of Alt and Base, calculated within repetitions and averaged across repetitions. These averaged relative differences were calculated for each genetic effect (within bars) or for the breeding goal (above the stacked bars).as the Initialization phase but applied EBV-based selection, and (2) the Alternative scheme, which also used EBV-based selection but shortened the dam generation interval to one year, phenotyping only the early trait on potential dams.

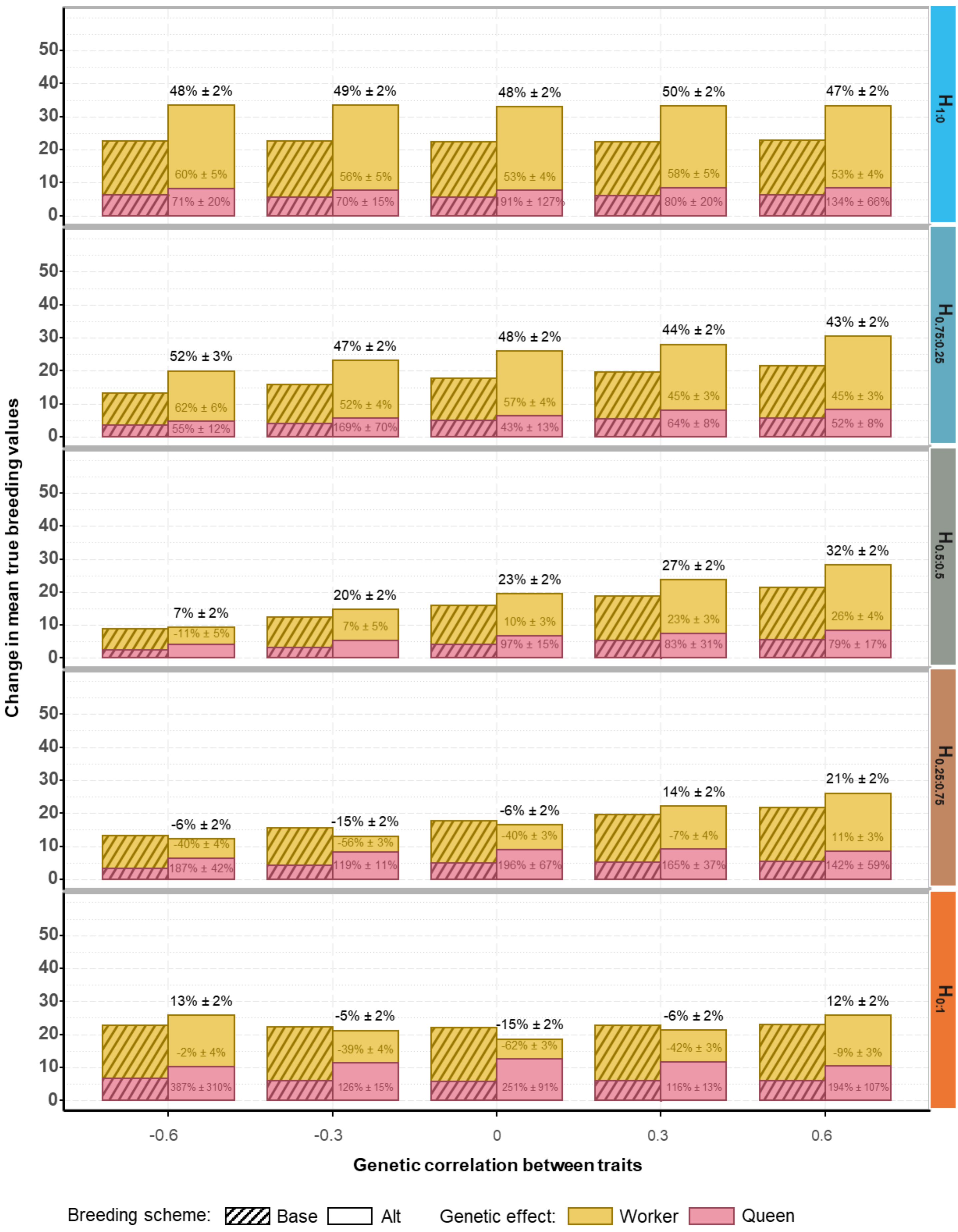

Fig. S3 Change in mean true breeding values since the end of Initialization and their relative difference between Alt and Base per effect and for the breeding goal after 20 years, for scenarios with a genetic correlation between worker and queen effects within a trait of -0.5.

H: breeding goal. The first number in the subscript refers to the weight on the early trait and the second number the weight on the late trait.
Base: reference breeding scheme will complete phenotyping and a 2-years generation interval on both the dam and the sire path.
Alt: accelerated breeding scheme in which only the early trait is phenotyped on potential dams, while potential sires are also phenotyped on the late trait. Partial phenotyping of the dams enables halving the dam generation interval to 1 year.
Breeding values are calculated as the change since the end of the Initialization phase and after 20 years of selection following the Base or Alt breeding scheme.
Text values shown represent the relative differences between the outcomes of Alt and Base, calculated within repetitions and averaged across repetitions. These averaged relative differences were calculated for each genetic effect (within bars) or for the breeding goal (above the stacked bars).as the Initialization phase but applied EBV-based selection, and (2) the Alternative scheme, which also used EBV-based selection but shortened the dam generation interval to one year, phenotyping only the early trait on potential dams.

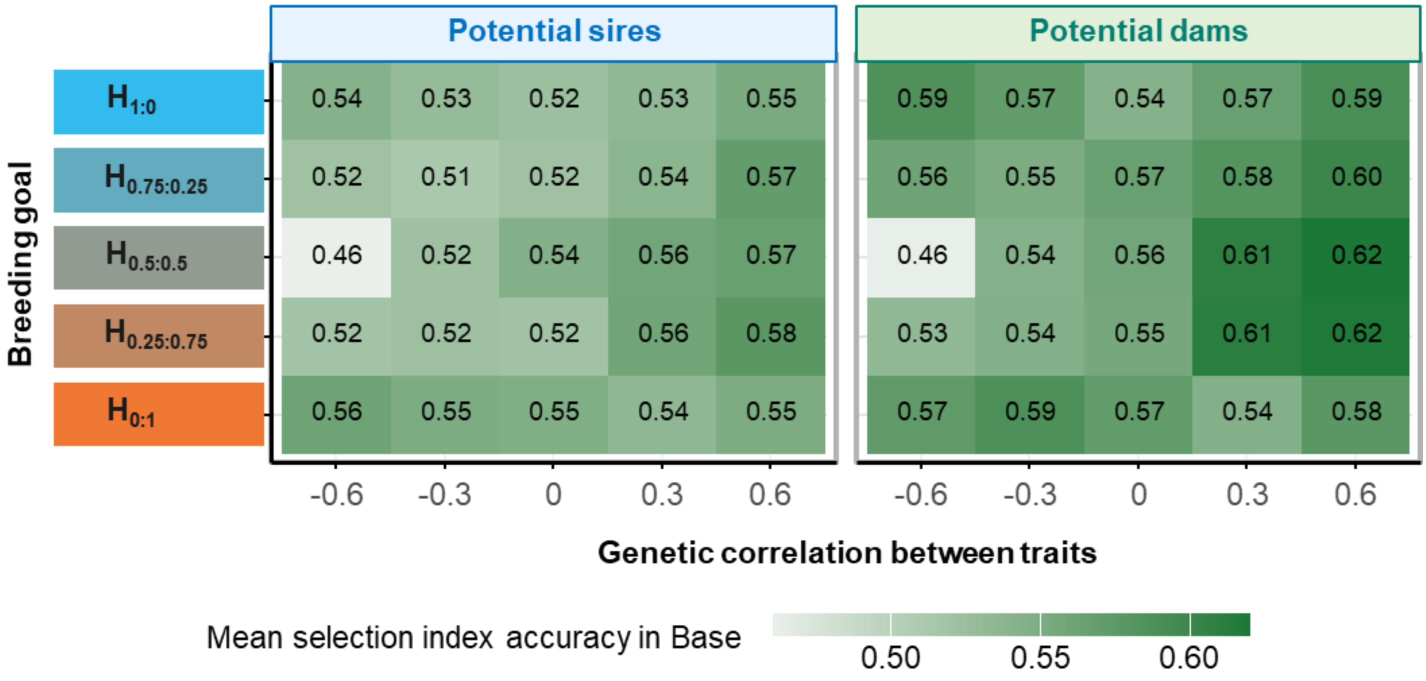

Supplementary Fig. S4 Mean selection index accuracy in the Base breeding scheme for potential sires and potential dams, and for scenarios with a null genetic correlation between worker and queen effects within a trait.

Base: reference breeding scheme will complete phenotyping and a 2-years generation interval on both the dam and the sire path.

The mean selection index accuracy was calculated as the correlation coefficient between the true and the estimated selection index averaged across repetitions of a scenario.

Values shown are for the last year of simulation (year 30).

Standard errors of the mean selection accuracy varied from 0.01 to 0.02.

H: breeding goal. The first number in the subscript refers to the weight on the early trait and the second number the weight on the late trait.

Supplementary Tables

Genetic gain

Table S1: Change in the mean true breeding values for the breeding goal since the end of Initialization and relative difference in the Base and Alt breeding scheme, for all scenarios with a null genetic correlation between worker and queen effects within a trait.

Breeding goal	$r_{T1,T2}$	Added genetic values for H		Relative difference between both breeding schemes (%)
		Base breeding scheme	Alt breeding scheme	
$H_{1:0}$	0	34.91 (0.39)	51.56 (0.41)	48.45 (1.79)
	-0.3	33.92 (0.36)	51.79 (0.37)	53.43 (1.78)
	-0.6	35.12 (0.37)	52.25 (0.42)	49.57 (1.93)
	0.3	34.99 (0.30)	52.49 (0.49)	50.61 (2.00)
	0.6	35.45 (0.31)	52.54 (0.45)	48.58 (1.46)
$H_{0.75:0.25}$	0	27.15 (0.24)	39.81 (0.25)	47.19 (1.58)
	-0.3	24.57 (0.22)	36.06 (0.32)	47.21 (1.65)
	-0.6	21.31 (0.24)	31.20 (0.28)	47.22 (1.93)
	0.3	30.04 (0.25)	44.05 (0.38)	47.06 (1.58)
	0.6	33.57 (0.29)	48.51 (0.37)	44.99 (1.61)
$H_{0.5:0.5}$	0	25.03 (0.24)	31.89 (0.28)	27.97 (1.60)
	-0.3	19.75 (0.22)	24.37 (0.25)	24.22 (2.06)
	-0.6	13.64 (0.22)	16.04 (0.26)	18.74 (2.42)
	0.3	28.59 (0.28)	38.36 (0.35)	34.65 (1.57)
	0.6	32.54 (0.27)	45.11 (0.38)	38.94 (1.39)
$H_{0.25:0.75}$	0	27.19 (0.29)	28.78 (0.40)	6.28 (1.67)
	-0.3	23.59 (0.24)	23.44 (0.36)	-0.44 (1.49)
	-0.6	21.43 (0.22)	21.81 (0.28)	2.32 (1.72)
	0.3	30.35 (0.25)	36.11 (0.33)	19.20 (1.18)
	0.6	33.80 (0.30)	42.70 (0.39)	26.60 (1.20)
$H_{0:1}$	0	34.90 (0.34)	33.02 (0.54)	-5.18 (1.52)
	-0.3	34.90 (0.36)	35.91 (0.65)	3.17 (1.87)
	-0.6	35.84 (0.33)	42.81 (0.49)	19.76 (1.51)
	0.3	34.84 (0.39)	35.66 (0.56)	2.85 (1.87)
	0.6	35.47 (0.43)	42.62 (0.37)	20.82 (1.49)
**Average**		29.72 (0.29)	38.36 (0.39)	28.97 (1.67)

H: breeding goal. The first number in the subscript refers to the weight on the early trait and the second number the weight on the late trait.
$r_{T1,T2}$: genetic correlation between the early and late trait.
Base: reference breeding scheme with complete phenotyping and a 2-years generation interval on both the dam and the sire path.
Alt: accelerated breeding scheme in which only the early trait is phenotyped on potential dams, while potential sires are also phenotyped on the late trait. Partial phenotyping of the dams enables halving the dam generation interval to 1 year.

Table S2: Change in the mean true breeding values for the breeding goal since the end of Initialization and relative difference in the Base and Alt breeding scheme, for all scenarios with a genetic correlation between worker and queen effects within a trait of -0.5.

Breeding goal	$r_{T1,T2}$	Added genetic values for H		Relative difference between both breeding schemes (%)
		Base breeding scheme	Alt breeding scheme	
$H_{1:0}$	0	22.41 (0.29)	32.91 (0.30)	47.80 (1.80)
	-0.3	22.61 (0.26)	33.46 (0.28)	49.02 (2.14)
	-0.6	22.72 (0.26)	33.45 (0.40)	47.95 (2.11)
	0.3	22.33 (0.30)	33.29 (0.37)	50.20 (2.33)
	0.6	22.91 (0.30)	33.33 (0.29)	46.64 (2.13)
$H_{0.75:0.25}$	0	17.72 (0.24)	26.01 (0.24)	48.01 (2.12)
	-0.3	15.81 (0.19)	23.11 (0.23)	47.11 (2.00)
	-0.6	13.30 (0.21)	19.95 (0.20)	52.07 (3.12)
	0.3	19.51 (0.23)	28.01 (0.27)	44.50 (2.05)
	0.6	21.41 (0.26)	30.50 (0.32)	43.32 (2.04)
$H_{0.5:0.5}$	0	15.85 (0.19)	19.40 (0.26)	23.07 (1.91)
	-0.3	12.37 (0.20)	14.72 (0.23)	20.12 (2.29)
	-0.6	8.75 (0.16)	9.23 (0.17)	6.86 (2.26)
	0.3	18.84 (0.22)	23.81 (0.24)	27.14 (1.74)
	0.6	21.39 (0.26)	28.12 (0.32)	32.18 (1.88)
$H_{0.25:0.75}$	0	17.66 (0.25)	16.53 (0.30)	-5.80 (1.85)
	-0.3	15.47 (0.21)	13.08 (0.28)	-14.59 (2.12)
	-0.6	13.27 (0.19)	12.32 (0.23)	-6.35 (2.08)
	0.3	19.61 (0.21)	22.16 (0.33)	13.60 (1.91)
	0.6	21.69 (0.21)	26.07 (0.35)	20.87 (2.01)
$H_{0:1}$	0	22.15 (0.30)	18.59 (0.39)	-15.45 (1.91)
	-0.3	22.29 (0.30)	21.19 (0.46)	-4.53 (2.05)
	-0.6	22.85 (0.27)	25.77 (0.39)	13.35 (1.88)
	0.3	22.73 (0.24)	21.27 (0.41)	-6.15 (1.84)
	0.6	23.08 (0.29)	25.71 (0.37)	12.06 (1.89)
**Average**		19.15 (0.24)	23.68 (0.31)	23.72 (2.06)

H: breeding goal. The first number in the subscript refers to the weight on the early trait and the second number the weight on the late trait.
$r_{T1,T2}$: genetic correlation between the early and late trait.
Base: reference breeding scheme with complete phenotyping and a 2-years generation interval on both the dam and the sire path.
Alt: accelerated breeding scheme in which only the early trait is phenotyped on potential dams, while potential sires are also phenotyped on the late trait. Partial phenotyping of the dams enables halving the dam generation interval to 1 year.

Inbreeding coefficients

Table S3: Increase in the mean inbreeding coefficient (%) since the end of Initialization and relative difference in the Base and Alt breeding scheme, for all scenarios with a null genetic correlation between worker and queen effects within a trait.

Breeding goal	$r_{T1,T2}$	Added inbreeding coefficient (%)		Relative difference between both breeding schemes (%)
		Base breeding scheme	Alt breeding scheme	
$H_{1:0}$	0	22.75 (0.48)	27.69 (0.41)	24.31 (3.17)
	-0.3	23.97 (0.54)	27.49 (0.47)	17.23 (3.09)
	-0.6	22.42 (0.37)	27.24 (0.37)	22.77 (2.28)
	0.3	23.96 (0.44)	27.60 (0.35)	17.20 (2.74)
	0.6	21.79 (0.55)	26.74 (0.43)	25.93 (3.31)
$H_{0.75:0.25}$	0	22.56 (0.55)	26.83 (0.40)	22.12 (3.01)
	-0.3	22.91 (0.43)	26.94 (0.40)	19.13 (2.44)
	-0.6	23.12 (0.38)	27.49 (0.44)	20.09 (2.42)
	0.3	22.95 (0.48)	27.20 (0.46)	20.75 (2.84)
	0.6	22.39 (0.39)	26.47 (0.35)	19.97 (2.54)
$H_{0.5:0.5}$	0	23.72 (0.55)	28.20 (0.40)	21.78 (3.03)
	-0.3	23.62 (0.48)	27.92 (0.45)	20.34 (2.80)
	-0.6	24.32 (0.51)	28.51 (0.38)	19.57 (2.79)
	0.3	23.05 (0.36)	28.27 (0.36)	23.91 (2.27)
	0.6	22.32 (0.52)	27.21 (0.43)	24.46 (2.92)
$H_{0.25:0.75}$	0	23.72 (0.46)	28.80 (0.41)	23.63 (2.98)
	-0.3	23.49 (0.46)	29.21 (0.39)	26.50 (2.80)
	-0.6	23.17 (0.56)	29.28 (0.46)	29.16 (3.27)
	0.3	23.02 (0.44)	28.45 (0.47)	25.12 (2.67)
	0.6	22.98 (0.51)	27.30 (0.43)	21.69 (3.33)
$H_{0:1}$	0	23.23 (0.43)	30.11 (0.50)	31.23 (2.65)
	-0.3	23.80 (0.45)	29.24 (0.42)	24.53 (2.44)
	-0.6	22.67 (0.46)	28.19 (0.40)	26.96 (3.34)
	0.3	23.25 (0.49)	28.42 (0.39)	24.64 (2.91)
	0.6	23.03 (0.46)	28.30 (0.44)	24.58 (2.46)
**Average**		23.13 (0.47)	27.96 (0.42)	23.10 (2.82)

H: breeding goal. The first number in the subscript refers to the weight on the early trait and the second number the weight on the late trait.
$r_{T1,T2}$: genetic correlation between the early and late trait.
Base: reference breeding scheme with complete phenotyping and a 2-years generation interval on both the dam and the sire path.
Alt: accelerated breeding scheme in which only the early trait is phenotyped on potential dams, while potential sires are also phenotyped on the late trait. Partial phenotyping of the dams enables halving the dam generation interval to 1 year.

Table S4: Increase in mean inbreeding coefficient (%) since the end of Initialization and relative difference in the Base and Alt breeding scheme, for all scenarios with a genetic correlation between worker and queen effects within a trait of -0.5.

Breeding goal	$r_{T1,T2}$	Added inbreeding coefficient (%)		Relative difference between both breeding schemes (%)
		Base breeding scheme	Alt breeding scheme	
$H_{1:0}$	0	24.38 (0.46)	29.21 (0.45)	21.74 (2.68)
	-0.3	24.56 (0.52)	28.96 (0.41)	20.29 (2.63)
	-0.6	23.64 (0.43)	28.28 (0.38)	21.38 (2.44)
	0.3	23.73 (0.44)	29.48 (0.46)	26.11 (2.73)
	0.6	23.54 (0.41)	28.05 (0.37)	20.99 (2.63)
$H_{0.75:0.25}$	0	23.78 (0.47)	28.24 (0.39)	20.91 (2.59)
	-0.3	23.92 (0.45)	28.75 (0.43)	22.19 (2.72)
	-0.6	23.35 (0.49)	29.15 (0.38)	27.27 (2.73)
	0.3	24.41 (0.48)	28.82 (0.34)	20.31 (2.55)
	0.6	23.33 (0.47)	28.54 (0.44)	24.04 (2.53)
$H_{0.5:0.5}$	0	24.31 (0.53)	28.57 (0.40)	20.28 (2.78)
	-0.3	24.30 (0.48)	28.76 (0.42)	20.64 (2.82)
	-0.6	25.15 (0.47)	29.38 (0.39)	18.66 (2.53)
	0.3	24.35 (0.42)	29.30 (0.44)	21.59 (2.15)
	0.6	23.82 (0.42)	28.65 (0.50)	21.55 (2.41)
$H_{0.25:0.75}$	0	23.93 (0.43)	28.76 (0.33)	21.80 (2.10)
	-0.3	23.71 (0.51)	29.71 (0.34)	28.43 (3.17)
	-0.6	24.44 (0.48)	29.62 (0.44)	23.16 (2.71)
	0.3	24.02 (0.46)	29.06 (0.52)	22.72 (2.79)
	0.6	24.24 (0.36)	28.60 (0.43)	19.13 (2.33)
$H_{0:1}$	0	23.45 (0.47)	30.31 (0.38)	32.00 (3.08)
	-0.3	24.15 (0.39)	29.54 (0.39)	24.07 (2.59)
	-0.6	24.26 (0.50)	28.97 (0.38)	21.74 (2.70)
	0.3	24.20 (0.50)	29.18 (0.37)	22.96 (2.65)
	0.6	24.07 (0.53)	28.27 (0.43)	19.75 (2.75)
**Average**		24.04 (0.46)	28.97 (0.41)	22.55 (2.63)

H: breeding goal. The first number in the subscript refers to the weight on the early trait and the second number the weight on the late trait.
$r_{T1,T2}$: genetic correlation between the early and late trait.
Base: reference breeding scheme with complete phenotyping and a 2-years generation interval on both the dam and the sire path.
Alt: accelerated breeding scheme in which only the early trait is phenotyped on potential dams, while potential sires are also phenotyped on the late trait. Partial phenotyping of the dams enables halving the dam generation interval to 1 year.

Inbreeding rate per generation

Table S5: Inbreeding rate per generation and relative difference in the Base and Alt breeding scheme for all scenarios with a null genetic correlation between worker and queen effects within a trait.

Breeding goal	$r_{T1,T2}$	Inbreeding rate per generation (%)		Relative difference between both breeding schemes (%)
		Base breeding scheme	Alt breeding scheme	
$H_{1:0}$	0	2.28 (0.05)	2.08 (0.03)	-6.79 (2.38)
	-0.3	2.40 (0.05)	2.06 (0.03)	-12.09 (2.32)
	-0.6	2.24 (0.04)	2.04 (0.03)	-7.94 (1.72)
	0.3	2.39 (0.04)	2.07 (0.03)	-12.10 (2.05)
	0.6	2.18 (0.05)	2.01 (0.03)	-5.58 (2.48)
$H_{0.75:0.25}$	0	2.29 (0.06)	2.02 (0.03)	-9.44 (2.32)
	-0.3	2.29 (0.04)	2.02 (0.03)	-10.69 (1.83)
	-0.6	2.31 (0.04)	2.06 (0.03)	-9.92 (1.81)
	0.3	2.29 (0.05)	2.04 (0.03)	-9.41 (2.12)
	0.6	2.24 (0.04)	1.99 (0.03)	-10.01 (1.90)
$H_{0.5:0.5}$	0	2.39 (0.05)	2.11 (0.03)	-9.22 (2.27)
	-0.3	2.36 (0.05)	2.09 (0.03)	-9.70 (2.10)
	-0.6	2.43 (0.05)	2.14 (0.03)	-10.31 (2.10)
	0.3	2.31 (0.04)	2.12 (0.03)	-7.12 (1.72)
	0.6	2.23 (0.05)	2.04 (0.03)	-6.61 (2.19)
$H_{0.25:0.75}$	0	2.37 (0.05)	2.16 (0.03)	-7.30 (2.24)
	-0.3	2.35 (0.05)	2.19 (0.03)	-5.06 (2.11)
	-0.6	2.32 (0.06)	2.20 (0.03)	-3.09 (2.45)
	0.3	2.30 (0.04)	2.14 (0.04)	-6.20 (1.99)
	0.6	2.30 (0.05)	2.05 (0.03)	-8.80 (2.50)
$H_{0:1}$	0	2.33 (0.04)	2.26 (0.04)	-1.68 (1.99)
	-0.3	2.38 (0.05)	2.19 (0.03)	-6.59 (1.83)
	-0.6	2.29 (0.05)	2.11 (0.03)	-5.75 (2.42)
	0.3	2.33 (0.05)	2.13 (0.03)	-6.53 (2.18)
	0.6	2.30 (0.05)	2.12 (0.03)	-6.58 (1.84)
**Average**		2.32 (0.05)	2.10 (0.03)	-7.78 (2.11)

H: breeding goal. The first number in the subscript refers to the weight on the early trait and the second number the weight on the late trait.
$r_{T1,T2}$: genetic correlation between the early and late trait.
Base: reference breeding scheme with complete phenotyping and a 2-years generation interval on both the dam and the sire path.
Alt: accelerated breeding scheme in which only the early trait is phenotyped on potential dams, while potential sires are also phenotyped on the late trait. Partial phenotyping of the dams enables halving the dam generation interval to 1 year.

Table S6: Inbreeding rate per generation and relative difference in the Base and Alt breeding scheme, for all scenarios with a genetic correlation between worker and queen effects within a trait of -0.5.

Breeding goal	$r_{T1,T2}$	Inbreeding rate per generation (%)		Relative difference between both breeding schemes (%)
		Base breeding scheme	Alt breeding scheme	
$H_{1:0}$	0	2.44 (0.05)	2.19 (0.03)	-8.68 (2.00)
	-0.3	2.46 (0.05)	2.17 (0.03)	-9.77 (1.96)
	-0.6	2.36 (0.04)	2.12 (0.03)	-8.92 (1.83)
	0.3	2.37 (0.04)	2.21 (0.03)	-5.43 (2.05)
	0.6	2.35 (0.04)	2.10 (0.03)	-9.22 (1.96)
$H_{0.75:0.25}$	0	2.37 (0.05)	2.12 (0.03)	-9.30 (1.94)
	-0.3	2.39 (0.04)	2.16 (0.03)	-8.36 (2.05)
	-0.6	2.33 (0.05)	2.19 (0.03)	-4.50 (2.06)
	0.3	2.44 (0.05)	2.16 (0.03)	-9.76 (1.91)
	0.6	2.33 (0.05)	2.14 (0.03)	-6.89 (1.91)
$H_{0.5:0.5}$	0	2.43 (0.05)	2.14 (0.03)	-9.82 (2.09)
	-0.3	2.43 (0.05)	2.16 (0.03)	-9.50 (2.12)
	-0.6	2.52 (0.05)	2.20 (0.03)	-11.01 (1.89)
	0.3	2.43 (0.04)	2.20 (0.03)	-8.81 (1.62)
	0.6	2.38 (0.04)	2.15 (0.04)	-8.84 (1.81)
$H_{0.25:0.75}$	0	2.39 (0.04)	2.16 (0.02)	-8.60 (1.57)
	-0.3	2.37 (0.05)	2.23 (0.03)	-3.60 (2.39)
	-0.6	2.44 (0.05)	2.22 (0.03)	-7.59 (2.04)
	0.3	2.40 (0.05)	2.18 (0.04)	-7.97 (2.08)
	0.6	2.42 (0.04)	2.14 (0.03)	-10.66 (1.73)
$H_{0:1}$	0	2.35 (0.05)	2.27 (0.03)	-1.07 (2.31)
	-0.3	2.42 (0.04)	2.22 (0.03)	-7.01 (1.93)
	-0.6	2.43 (0.05)	2.17 (0.03)	-8.76 (2.01)
	0.3	2.42 (0.05)	2.19 (0.03)	-7.78 (1.98)
	0.6	2.41 (0.05)	2.12 (0.03)	-10.20 (2.06)
**Average**		2.10 (0.05)	2.17 (0.03)	-8.08 (1.97)

H: breeding goal. The first number in the subscript refers to the weight on the early trait and the second number the weight on the late trait.
$r_{T1,T2}$: genetic correlation between the early and late trait.
Base: reference breeding scheme with complete phenotyping and a 2-years generation interval on both the dam and the sire path.
Alt: accelerated breeding scheme in which only the early trait is phenotyped on potential dams, while potential sires are also phenotyped on the late trait. Partial phenotyping of the dams enables halving the dam generation interval to 1 year.

Inbreeding rate per generation in 36 BQs scenarios and comparison to 24 BQs scenarios

Table S7: Inbreeding rate per generation in 36 BQs scenarios in the Base and Alt breeding scheme and relative difference with 24 BQs scenarios, and a null genetic correlation between worker and queen effects within a trait.

Breeding goal	$r_{T1,T2}$	Inbreeding rate per generation in 36 BQs scenarios (%)		Relative difference between 36 and 24 BQs scenarios (%)		Relative difference between 36 BQs scenarios in Alt and 24 BQs scenarios in Base (%)
		Base breeding scheme	Alt breeding scheme	Base breeding scheme	Alt breeding scheme	
$H_{1:0}$	0	1.53 (0.03)	1.48 (0.02)	-32.75 (2.01)	-28.61 (1.57)	-13.13 (2.33)
	-0.6	1.55 (0.03)	1.37 (0.02)	-31.07 (1.86)	-32.93 (1.49)	-18.51 (1.97)
	0.6	1.52 (0.03)	1.45 (0.02)	-30.37 (2.26)	-27.64 (1.53)	-11.21 (2.55)
$H_{0.75:0.25}$	0	1.51 (0.03)	1.43 (0.02)	-34.16 (2.14)	-29.42 (1.57)	-16.88 (2.39)
	-0.6	1.57 (0.03)	1.43 (0.02)	-31.87 (1.74)	-30.61 (1.46)	-17.50 (1.75)
	0.6	1.52 (0.03)	1.42 (0.02)	-32.07 (1.82)	-28.33 (1.39)	-15.27 (1.91)
$H_{0.5:0.5}$	0	1.60 (0.03)	1.49 (0.03)	-32.78 (2.04)	-29.46 (1.57)	-16.68 (2.40)
	-0.6	1.66 (0.03)	1.49 (0.02)	-31.78 (1.97)	-30.16 (1.42)	-18.12 (2.14)
	0.6	1.52 (0.03)	1.40 (0.02)	-31.98 (2.10)	-31.40 (1.63)	-16.37 (2.44)
$H_{0.25:0.75}$	0	1.58 (0.04)	1.64 (0.03)	-33.34 (2.01)	-24.09 (1.73)	-7.82 (2.42)
	-0.6	1.59 (0.03)	1.58 (0.03)	-31.23 (2.04)	-28.08 (1.62)	-9.13 (2.62)
	0.6	1.49 (0.04)	1.46 (0.03)	-34.99 (2.10)	-28.93 (1.76)	-15.57 (2.47)
$H_{0:1}$	0	1.60 (0.03)	1.65 (0.02)	-30.99 (1.72)	-26.75 (1.59)	-5.04 (2.21)
	-0.6	1.56 (0.03)	1.46 (0.02)	-32.02 (2.01)	-30.88 (1.49)	-14.85 (2.26)
	0.6	1.52 (0.03)	1.47 (0.02)	-34.04 (1.83)	-30.78 (1.49)	-14.95 (2.13)

In the 36 BQs scenarios, compared to the 24 BQs scenario, the number of queens in the breeding nucleus has been increase by 50% but the candidate population remained the same (lowering the selection intensity).
H: breeding goal. The first number in the subscript refers to the weight on the early trait and the second number the weight on the late trait.
rT1,T2: genetic correlation between the early and late trait.
Base: reference breeding scheme with complete phenotyping and a 2-years generation interval on both the dam and the sire path.
Alt: accelerated breeding scheme in which only the early trait is phenotyped on potential dams, while potential sires are also phenotyped on the late trait. Partial phenotyping of the dams enables halving the dam generation interval to 1 year.
Standard errors (SE) are shown in brackets. SE for relative differences (SE_R) across 24 and 36 BQs simulations were estimated using propagation of uncertainties from SE of means (μ) obtained within 24 (SE_{24}) and within 36 BQs (SE_{36}) simulations:

$$SE_R = \frac{1}{\mu_{24}}\sqrt{SE_{36}^2 + \left(\frac{\mu_{36} \cdot SE_{24}}{\mu_{24}}\right)^2}$$

Table S8: Inbreeding rate per generation in 36 BQs scenarios in the Base and Alt breeding scheme and relative difference with 24 BQs scenarios, and a genetic correlation between worker and queen effects within a trait of -0.5.

Breeding goal	$r_{T1,T2}$	Inbreeding rate in 36 BQs scenarios (%)		Relative difference between 36 and 24 BQs scenarios (%)		Relative difference between 36 BQs scenarios in Alt and 24 BQs scenarios in Base (%)
		Base breeding scheme	Alt breeding scheme	Base breeding scheme	Alt breeding scheme	
$H_{1:0}$	0	1.64 (0.04)	1.53 (0.03)	-32.61 (2.01)	-30.10 (1.65)	-16.28 (2.20)
	-0.6	1.69 (0.03)	1.54 (0.03)	-28.49 (1.91)	-27.48 (1.54)	-13.24 (2.14)
	0.6	1.66 (0.03)	1.50 (0.02)	-29.65 (1.83)	-28.80 (1.50)	-15.15 (2.02)
$H_{0.75:0.25}$	0	1.74 (0.04)	1.55 (0.03)	-27.02 (2.12)	-26.66 (1.67)	-12.87 (2.33)
	-0.6	1.58 (0.04)	1.50 (0.03)	-32.53 (2.13)	-31.18 (1.48)	-14.06 (2.32)
	0.6	1.65 (0.03)	1.52 (0.03)	-29.46 (2.03)	-29.17 (1.64)	-13.37 (2.29)
$H_{0.5:0.5}$	0	1.62 (0.04)	1.55 (0.02)	-33.22 (2.08)	-27.88 (1.50)	-15.21 (2.27)
	-0.6	1.69 (0.03)	1.62 (0.03)	-32.82 (1.69)	-26.60 (1.69)	-14.26 (2.27)
	0.6	1.58 (0.04)	1.51 (0.03)	-33.86 (2.02)	-29.61 (1.69)	-15.33 (2.07)
$H_{0.25:0.75}$	0	1.63 (0.04)	1.57 (0.03)	-31.82 (1.94)	-27.28 (1.53)	-12.57 (2.21)
	-0.6	1.68 (0.03)	1.64 (0.03)	-31.09 (1.97)	-26.26 (1.61)	-10.65 (2.27)
	0.6	1.62 (0.03)	1.53 (0.02)	-33.30 (1.74)	-28.52 (1.45)	-15.67 (1.70)
$H_{0:1}$	0	1.63 (0.03)	1.66 (0.03)	-30.48 (2.03)	-26.84 (1.55)	-5.45 (2.48)
	-0.6	1.69 (0.04)	1.54 (0.03)	-30.30 (2.18)	-28.89 (1.50)	-15.10 (2.25)
	0.6	1.61 (0.03)	1.49 (0.03)	-33.11 (2.06)	-29.94 (1.63)	-17.71 (2.32)

In the 36 BQs scenarios, compared to the 24 BQs scenario, the number of queens in the breeding nucleus has been increase by 50% but the candidate population remained the same (lowering the selection intensity).
H: breeding goal. The first number in the subscript refers to the weight on the early trait and the second number the weight on the late trait.
rT1,T2: genetic correlation between the early and late trait.
Base: reference breeding scheme with complete phenotyping and a 2-years generation interval on both the dam and the sire path.
Alt: accelerated breeding scheme in which only the early trait is phenotyped on potential dams, while potential sires are also phenotyped on the late trait. Partial phenotyping of the dams enables halving the dam generation interval to 1 year.
Standard errors (SE) are shown in brackets. SE for relative differences (SE_R) across 24 and 36 BQs simulations were estimated using propagation of uncertainties from SE of means (μ) obtained within 24 (SE_{24}) and within 36 BQs (SE_{36}) simulations:

$$SE_R = \frac{1}{\mu_{24}}\sqrt{SE_{36}^2 + \left(\frac{\mu_{36} \cdot SE_{24}}{\mu_{24}}\right)^2}$$

Genetic gain in 36 BQs scenarios and comparison to 24 BQs scenarios

Table S9: Increase in genetic value for the breeding goal since the end of Initialization in 36 BQs scenarios in the Base and Alt breeding scheme and relative difference with 24 BQs scenarios, scenarios with a null genetic correlation between worker and queen effects within a trait.

Breeding goal	$r_{T1,T2}$	Genetic gain in 36 BQs scenarios (%)		Relative difference between 36 and 24 BQs scenarios (%)		Relative difference between 36 BQs scenarios in Alt and 24 BQs scenarios in Base (%)
		Base breeding scheme	Alt breeding scheme	Base breeding scheme	Alt breeding scheme	
$H_{1:0}$	0	31.57 (0.29)	49.31 (0.30)	-9.58 (1.31)	-4.35 (0.96)	41.25 (1.80)
	-0.6	32.85 (0.30)	49.56 (0.30)	-6.46 (1.03)	-5.15 (0.95)	41.13 (1.70)
	0.6	33.04 (0.28)	50.14 (0.36)	-6.79 (1.13)	-4.57 (1.07)	41.44 (1.60)
$H_{0.75:0.25}$	0	25.30 (0.20)	37.65 (0.29)	-6.82 (1.11)	-5.43 (0.94)	38.68 (1.65)
	-0.6	19.62 (0.21)	30.18 (0.18)	-7.92 (1.43)	-3.29 (1.03)	41.59 (1.82)
	0.6	31.16 (0.23)	46.17 (0.18)	-7.17 (1.06)	-4.82 (0.83)	37.54 (1.32)
$H_{0.5:0.5}$	0	22.83 (0.18)	30.70 (0.23)	-8.77 (1.12)	-3.75 (1.10)	22.65 (1.48)
	-0.6	12.90 (0.14)	15.44 (0.18)	-5.48 (1.84)	-3.73 (1.93)	13.17 (2.29)
	0.6	30.32 (0.20)	42.86 (0.29)	-6.82 (0.98)	-4.99 (1.03)	31.70 (1.40)
$H_{0.25:0.75}$	0	25.63 (0.22)	28.95 (0.32)	-5.73 (1.28)	0.57 (1.80)	6.46 (1.64)
	-0.6	20.48 (0.21)	22.52 (0.29)	-4.45 (1.38)	3.25 (1.88)	5.09 (1.72)
	0.6	30.98 (0.25)	40.81 (0.33)	-8.36 (1.10)	-4.43 (1.16)	20.72 (1.46)
$H_{0:1}$	0	32.19 (0.27)	34.11 (0.48)	-7.75 (1.19)	3.30 (2.23)	-2.24 (1.68)
	-0.6	33.30 (0.29)	40.86 (0.33)	-7.08 (1.17)	-4.55 (1.34)	13.99 (1.39)
	0.6	33.31 (0.28)	40.63 (0.41)	-6.10 (1.38)	-4.67 (1.27)	14.56 (1.81)

In the 36 BQs scenarios, compared to the 24 BQs scenario, the number of queens in the breeding nucleus has been increase by 50% but the candidate population remained the same (lowering the selection intensity).
H: breeding goal. The first number in the subscript refers to the weight on the early trait and the second number the weight on the late trait.
rT1,T2: genetic correlation between the early and late trait.
Base: reference breeding scheme with complete phenotyping and a 2-years generation interval on both the dam and the sire path.
Alt: accelerated breeding scheme in which only the early trait is phenotyped on potential dams, while potential sires are also phenotyped on the late trait. Partial phenotyping of the dams enables halving the dam generation interval to 1 year.
Standard errors (SE) are shown in brackets. SE for relative differences (SE_R) across 24 and 36 BQs simulations were estimated using propagation of uncertainties from SE of means (μ) obtained within 24 (SE_{24}) and within 36 BQs (SE_{36}) simulations:

$$SE_R = \frac{1}{\mu_{24}} \sqrt{SE_{36}^2 + \left(\frac{\mu_{36} \cdot SE_{24}}{\mu_{24}}\right)^2}$$

Table S10: Increase in genetic value for the breeding goal since the end of Initialization in 36 BQs scenarios in the Base and Alt breeding scheme and relative difference with 24 BQs scenarios, and a genetic correlation between worker and queen effects within a trait of -0.5.

Breeding goal	$r_{T1,T2}$	Genetic gain in 36 BQs scenarios (%)		Relative difference between 36 and 24 BQs scenarios (%)		Relative difference between 36 BQs scenarios in Alt and 24 BQs scenarios in Base (%)
		Base breeding scheme	Alt breeding scheme	Base breeding scheme	Alt breeding scheme	
$H_{1:0}$	0	20.83 (0.25)	31.80 (0.35)	-7.05 (1.66)	-3.38 (1.38)	41.90 (2.44)
	-0.6	21.61 (0.26)	32.46 (0.30)	-4.87 (1.58)	-2.95 (1.46)	42.86 (2.11)
	0.6	22.04 (0.23)	32.22 (0.26)	-3.81 (1.62)	-3.34 (1.15)	40.62 (2.18)
$H_{0.75:0.25}$	0	16.54 (0.20)	24.57 (0.19)	-6.63 (1.68)	-5.51 (1.12)	38.70 (2.14)
	-0.6	12.76 (0.15)	19.53 (0.18)	-4.08 (1.88)	-2.08 (1.34)	46.82 (2.70)
	0.6	20.65 (0.19)	30.04 (0.23)	-3.56 (1.48)	-1.49 (1.28)	40.33 (2.03)
$H_{0.5:0.5}$	0	14.72 (0.17)	18.92 (0.27)	-7.12 (1.55)	-2.47 (1.91)	19.36 (2.23)
	-0.6	7.95 (0.14)	9.15 (0.17)	-9.10 (2.27)	-0.94 (2.55)	4.54 (2.69)
	0.6	19.67 (0.18)	27.23 (0.25)	-8.06 (1.41)	-3.16 (1.43)	27.30 (1.95)
$H_{0.25:0.75}$	0	16.98 (0.19)	17.08 (0.32)	-3.89 (1.72)	3.29 (2.70)	-3.30 (2.26)
	-0.6	12.68 (0.16)	12.95 (0.22)	-4.45 (1.80)	5.10 (2.70)	-2.47 (2.17)
	0.6	20.60 (0.23)	26.04 (0.25)	-4.99 (1.43)	-0.14 (1.64)	20.07 (1.64)
$H_{0:1}$	0	21.38 (0.25)	19.69 (0.40)	-3.49 (1.71)	5.92 (3.07)	-11.09 (2.16)
	-0.6	21.88 (0.25)	25.04 (0.30)	-4.22 (1.56)	-2.85 (1.87)	9.59 (1.83)
	0.6	21.62 (0.22)	25.08 (0.30)	-6.31 (1.51)	-2.42 (1.81)	8.70 (1.88)

In the 36 BQs scenarios, compared to the 24 BQs scenario, the number of queens in the breeding nucleus has been increase by 50% but the candidate population remained the same (lowering the selection intensity).
H: breeding goal. The first number in the subscript refers to the weight on the early trait and the second number the weight on the late trait.
rT1,T2: genetic correlation between the early and late trait.
Base: reference breeding scheme with complete phenotyping and a 2-years generation interval on both the dam and the sire path.
Alt: accelerated breeding scheme in which only the early trait is phenotyped on potential dams, while potential sires are also phenotyped on the late trait. Partial phenotyping of the dams enables halving the dam generation interval to 1 year.
Standard errors (SE) are shown in brackets. SE for relative differences (SE_R) across 24 and 36 BQs simulations were estimated using propagation of uncertainties from SE of means (μ) obtained within 24 (SE_{24}) and within 36 BQs (SE_{36}) simulations:

$$SE_R = \frac{1}{\mu_{24}} \sqrt{SE_{36}^2 + \left(\frac{\mu_{36} \cdot SE_{24}}{\mu_{24}}\right)^2}$$
